# Automatic or controlled: How does disbelief in free will influence cognitive functioning?

**DOI:** 10.1111/bjop.12578

**Published:** 2022-06-15

**Authors:** Maayan Katzir, Oliver Genschow

**Affiliations:** ^1^ Bar Ilan University Ramat Gan Israel; ^2^ University of Cologne Cologne Germany

**Keywords:** automaticity, cognitive control, free will belief, self‐control

## Abstract

Most people believe in free will. Past research has indicated that reducing this belief has numerous downstream consequences including everyday outcomes as well as neural and cognitive correlates associated with a reduction of self‐control. However, the exact mechanisms through which a reduction in free will belief affects self‐control are still a matter of investigation. In the present registered report, we used a task switching paradigm to examine whether reducing belief in free will makes people less controlled or whether it enhances their reliance on automatic impulses. Using Bayesian sequential analysis, we failed to conceptually replicate the previous link between free will belief and cognitive control. Our registered report plan mostly accumulated substantial evidence supporting the null hypothesis. That is, diminished belief in free will does neither impact control nor automaticity. Theoretical implications of this finding are discussed.

## INTRODUCTION

Although most people believe in free will (Baumeister et al., 2009; Nahmias et al., 2005), the question whether free will actually exists is a longstanding discussion in philosophy (Dennett, 2015; Van Inwagen, 1983). Recently, research in cognitive neuroscience has joined this debate by assaulting the plausibility of free will (Crick, 1994; Harris, 2012; Wegner, 2002). When such anti‐free will viewpoint became en vogue not only in academia, but also in the popular press (Chivers, 2010; Wolfe, 1997), psychologists started investigating the question whether believing in free will has implications for human behaviour (Rigoni & Brass, 2014; Shariff et al., 2008; Shariff & Vohs, 2014).

When assessing belief in free will, researchers typically study laypeople's concepts of free will (e.g., Nichols, 2006). These concepts can be defined as metacognitive judgements about the degree to which individuals intentionally guide their thoughts and actions (Frith, 2012). In this sense, laypeople's belief in free will reflects their perception that people are responsible for their actions (Carey & Paulhus, 2013) and that they can control their own behaviour (Paulhus & Carey, 2011).

Psychological research indicated that believing in free will has several downstream consequences. For instance, correlational data suggest that people who believe in free will perform better in a variety of contexts, such as in work (Stillman et al., 2010) and academic settings (Feldman et al., 2016). Believing in free will was also associated with intolerance of unethical behaviour and support for criminal punishment (Martin et al., 2017) as well as perceiving intentions in others (Genschow et al., 2019). Other researchers used manipulations such as having participants read a scientific text that denies the plausibility of free will in order to weaken participants' belief in free will. When weakening individuals' belief in free will by such manipulations, researchers found increased antisocial behaviour, such as racism (Zhao et al., 2014), aggressiveness towards others as well as decreased prosocial attitudes expressed in altruistic (Baumeister et al., 2009) and cooperative behaviour (Protzko et al., 2016), but also very basic effects on person perception (Genschow et al., 2017).

Interestingly, cognitive neuroscientific research suggests that such behavioural changes stemming from reduced free will beliefs may be linked to neural and cognitive mechanisms associated with self‐control (Rigoni et al., 2011, 2012, 2013, 2015). For example, Rigoni and colleagues (Rigoni et al., 2013) found that reading a scientific text that denies the existence of free will reduces the post error slowing effect – an effect associated with cognitive control that reflects appropriate behavioural adjustments (Botvinick et al., 2001; Kerns et al., 2004). Other research conceptually replicated this finding within a go no‐go task and demonstrated that anti‐free will belief manipulation reduces intentional inhibition (i.e., the decision to withhold a pre‐potent response) (Rigoni et al., 2012).

Despite these seminal findings, it is important to note that recent research applying free will belief manipulations found difficulties in replicating some of the landmark‐findings in this research area (Crone & Levy, 2019; Genschow et al., 2020; Giner‐Sorolla et al., 2016; Monroe et al., 2017; Open Science Collaboration, 2015; Schooler et al., 2014; Shariff & Vohs, 2014). For example, Monroe et al. (2017) could not find an effect of diminishing participants' belief in free will on moral behaviour, judgements of blame, or punishment decisions. More recently, Genschow et al. (2020) could not replicate the effect of manipulated belief in free will on punishment within a sample of professional judges. While these failed replications were mostly related to the relation of disbelief in free will and anti‐social behaviour and punishment, to the best of our knowledge there are no unsuccessful replications in the domain of self‐control. Nevertheless, as other downstream consequences from casting doubt on free will could fail to replicate, the question arises whether the link between disbelief in free will and self‐control can be replicated. Thus, we aimed at putting the self‐control hypothesis under a critical test. Moreover, although Rigoni and colleagues' (Rigoni et al., 2011, 2012, 2013, 2015) research demonstrates that anti‐free will belief manipulations influence cognitive control processes underlying self‐control, such research remains silent about the exact mechanisms through which disbelief in free will affects self‐control. Particularly, the question arises whether manipulating anti‐free will beliefs makes people less controlled or whether it enhances their reliance on automatic impulses. The aim of this research is to (1) conceptually replicate previous findings on the influence of disbelief on free will on self‐control and, if successful, to (2) pinpoint its underlying mechanisms.

### Controlled processes and automatic impulses

Dual process models of self‐control suggest that self‐control conflicts represent a conflict between the impulsive system (i.e., automatic tendencies), and the reflective control system (i.e., behaviours derived by the goal to self‐regulate) (Hofmann et al., 2009). According to this perspective, each system activates behavioural schemata. The strongest schemata are eventually executed thus determining behaviour. Diminished conflict, or improved self‐control, may reflect differences in the strength of activation of the impulsive system and the reflective control system. Because both processes (reduced automaticity vs. improved control) lead to the same outcome, traditional self‐control research is unable to differentiate between these processes.

The same problem applies when using measures of conflict. Measures of conflict are often seen as a marker for self‐control (Hofmann et al., 2012; Katzir et al., 2010; Muraven et al., 2006; Rigoni et al., 2015). However, they lack in pointing to the process underlying the conflict‐related outcome (Katzir, Ori, Eyal, et al., 2015a), because they could either be driven by control and/or automatic processes (see, for example, interpretations of the Stroop effect as a control index (Brown et al., 2007; Yeung & Monsell, 2003) vs. interpretations of it as an automaticity index (Huguet et al., 1999; Tzelgov, 1997). In order to disentangle these processes, which would allow for testing the actual process(es) underlying anti‐free will belief manipulations, the assessment of cognitive indices that are able to differentiate between control and automaticity is needed. In this respect, a promising venue is offered by task switching paradigms.

### Task switching paradigms

In typical task switching paradigms, participants classify stimuli according to two or more dimensions. The correct responses are defined by using stimulus–response sets (S‐R sets), which are an assembly of (usually two) stimulus–response rules (S‐R rules). For example, when classifying the colour of green and red circles and triangles, an S‐R set may be assembled by the S‐R rule ‘IF green THEN PRESS key 1’ and the S‐R rule ‘IF red THEN PRESS key 2’. Another S‐R set could be composed out of the S‐R rule ‘IF circle THEN PRESS key 1’ and the S‐R rule ‘IF triangle THEN PRESS key 2’. The stimuli and responses overlap between the tasks, and in a given trial a dimension can be either relevant (i.e., the instructed dimension for classification), or irrelevant. The overlap between responses and S‐R sets dictates that irrelevant S‐R rules can be either compatible (i.e., the same response is dictated by both the irrelevant and the relevant S‐R rules, e.g., when classifying the colour of a green circle) or competing (i.e., the response according to the irrelevant S‐R rule is different than the response according to the relevant S‐R rule, e.g., when classifying the colour of a green triangle). In such task switching paradigms, the congruency effect (Meiran & Kessler, 2008; Sudevan & Taylor, 1987) refers to the cost (either in Response Time – RT; or in Proportion of Errors – PE) when participants respond to incongruent trials (i.e., trials with at least one competing S‐R rule) compared to congruent trials (i.e., trials in which all irrelevant rules are compatible).

The congruency effect is generally seen as a conflict‐related index, because it represents a conflict between an automatic tendency (i.e., to react according to the competing S‐R rule) and a controlled tendency (i.e., to react according to the relevant rule indicated by the instructed dimension for classification) (Kiesel et al., 2007; Meiran & Kessler, 2008). The congruency effect may therefore represent interference that is posed by the potentiation of the currently irrelevant, and competing, S‐R rule(s). This interference would increase the congruency effect. However, controlled processes combating this interference would decrease the congruency effect. Therefore, for example, an enhanced congruency effect may reflect reduced control, or enhanced automaticity, or both. Thus, the congruency effect, similar to other self‐control outcome‐related measures (e.g., aggressiveness, prosocial behaviour, food intake) cannot differentiate between control and automaticity. To overcome this shortcoming, other indices afforded by task switching paradigms enable the assessment of aspects of cognitive control.

In this work, we focus on two crucial processes that allow differentiating control from automaticity – Backward Inhibition (BI, an index indicating inhibition of interfering stimulus response sets (Mayr & Keele, 2000) and Competitor Rule Suppression (CRS, an index indicating suppression of interfering response rules (Meiran et al., 2010) – both are considered relatively pure indices for cognitive control that do not involve automaticity (Hsieh et al., 2012; Katzir et al., 2018; Koch et al., 2010), but nonetheless represent different inhibitory processes (Regev & Meiran, 2016, 2017). We elaborate on each index in the Methods section. Importantly, these two indices are sequential effects, which measure only the trace of the cognitive control from Trial N‐1 that lingers to the following trial (Trial N). In both effects, this lingering control is manifested as performance cost in Trial N. Therefore, whereas enhanced cognitive control would be manifested in *reduced* congruency effect, it would be manifested as an *enhanced* Competitor Rule Suppression effect and an *enhanced* Backward Inhibition effect.

### Present research and hypotheses

Based on prior research connecting disbelief in free will to self‐control reduction, we hypothesize an association between diminished belief in free will and either reduced control or enhanced automaticity. Yet, because the focus of this investigation is on the exact underlying process (i.e., control vs. automaticity), we contrast two predictions.

*1) – Control‐Prediction*: If diminished belief in free will reduces controlled processes compared with belief in free will, we expect the anti‐free will belief manipulation to influence all the indices reviewed above (i.e., Congruency Effect, Backward Inhibition, and Competitor Rule Suppression). That is, compared to the pro‐free will group, in the anti‐free will group the Congruency Effect is expected to be enhanced, whereas Backward Inhibition and Competitor Rule Suppression are expected to be reduced.

*2 – Automaticity‐Prediction*: If diminished belief in free will enhances automaticity, the anti‐free will manipulation should influence *only* the congruency index, but not the two pure control indices, because the congruency effect is the only index that involves automaticity. More specifically, the Automaticity‐Prediction suggests that in the anti‐free will group, the congruency effect should be enhanced compared to the pro‐free will group. However, the anti‐free will belief manipulation should not influence the Backward Inhibition or the Competitor Rule Suppression indices because these indices are a relatively pure measure of control.

To sum up, we inspected two indices that involve only control (Backward Inhibition, Competitor Rule Suppression), and one index that involves both control and automaticity (Congruency Effect). If the Control‐Prediction is supported by the data, there is unequivocal evidence that disbelief in free will influences control, but we are unable to unequivocally rule out automaticity. If the Automaticity‐Prediction is supported by the data, we are able to conclude that automaticity, but not control, is influenced by disbelief in free will (see Katzir, Ori, Eyal, et al., 2015a for a similar rationalization).

## METHODS

### Participants

Participants (*N*_final_ = 204, 71 men, 132 women, and one preferred not to answer, age ranged from 18 to 73, *M*_age_ = 38.5, *SD*_age_ = 12.99) were recruited online via Prolific (see Peer et al., 2017, 2021, for the benefits of this participant pool). We compensated participants for their participation with £6.7. To assure the quality and reliability of our data, we followed several recommendations for online data collection put forward by Paolacci and Chandler (2014). First, we restricted participants in Prolific based on qualifications. Specifically, we restricted country of residence to the United Kingdom and first language to English. We also restricted participation to participants whose ratio of approved to submitted tasks is 95% or above. Although using experienced workers could potentially be problematic, this problem is circumvented by introducing a novel task, and, even if participants are familiarized with it, they cannot predict the study question. Second, as recommended by Paolacci and Chandler (2014), we also applied a performance contingent reward procedure, according to which participants were told that they need to ‘perform the study well’ to get a fixed bonus of £2 in addition to the payment of £4.7. In particular, we told participants that to perform well, ‘you need to read the instructions and follow them carefully. If you will simply click through the study, you will not be compensated at all’. Participants who completed the study and had less than 40% errors received the bonus.

#### Sampling procedure and data exclusion

In line with our preregistration (https://osf.io/8hx3w), we applied a Sequential Bayes Factor (SBF) design (Schönbrodt et al., 2017) with a minimal sample size of 60 participants in each experimental group, and a maximal sample size of 100 participants per experimental group. In a simulation (cf. Appendix S1 for the simulation code), we run with the BFDA R‐package (Schönbrodt, 2017; Schönbrodt & Wagenmakers, 2018) we followed Schönbrodt et al. (2017) recommendations and ran 10,000 simulated studies for H1 and additional 10,000 for H0, and 1 as Cauchy priors^1^ for independent sample *t*‐tests. We used the assumed effect size of Cohen's *d* = 0.5 (based on prior research on the effect of free will belief manipulations on self‐control), and the minimum (n.min) and maximum (n.max) sample size in each group of 60 (note that Schönbrodt and colleagues recommended a number of 20 participants per condition) and 100 (our budget limit), respectively.

As it is the case for most research projects, there is a trade‐off between high power and feasibility. To best solve this trade‐off, our goal was to minimize erroneous conclusions from the SBF. Although this goal can be easily achieved by increasing the maximal sample size, at some point this approach becomes not feasible anymore (Lakens, 2014). We therefore took the approach of reducing erroneous termination of the study. Thus, instead of starting with a minimal sample size of 20 participants in each group as recommended by Schönbrodt et al. (2017), we decided to increase the minimal sample (this decision is based on Schönbrodt et al.’s simulations as well as another simulation we ran, that indicated that most errors occur at a low sample size). Another measure we took to assure that our study does not terminate prematurely with an erroneous decision, was to set the boundaries of H1 and H0 to 10 (i.e., BF_10_ > 10) and 1/10 (i.e., BF_10_ < 1/10), respectively. Such boundaries are higher than recommended by Schönbrodt et al. (2017) for studies aiming to detect evidence in support of H0. In fact, Schönbrodt et al. (2017) recommended setting asymmetrical boundaries in such cases. Yet, facing the budgetary limitation of a total maximal sample of 200 participants (100 participants in each group), we decided that maintaining the high boundaries will assure the lowest erroneous pre‐mature terminations of study that we can afford. Nevertheless, if the study should terminate at n.max, we planned to use commonly accepted BFs interpretation norms (Jeffreys, 1961) to draw conclusions, as elaborated in the analyses section below. In line with Jeffreys (1961) we aimed at interpreting BFs >3 and BFs <1/3 as sufficient evidence for H1 and H0, respectively. BFs within the boundary 1/3 < BF <3, were planned to be interpreted as anecdotal evidence.

Our study had two goals. The first goal was to replicate previous findings (Rigoni et al., 2011, 2012, 2013, 2015) on the influence of disbelief in free will on cognitive control. The second goal was to understand the mechanism underlying this influence. For the replication purpose of our study, we ran a simulation with *Cohen's d* = 0.5 (as prior research on the effect of free will belief manipulations on self‐control yielded this effect), thus simulating a scenario in which H1 is true. In this simulation, 82.9% of the studies terminated in strong support for H1 (i.e., BF_10_ > 10), and only 0.1% of the studies terminated in strong support for H0 (i.e., BF_10_ < 1/10). Out of the remaining 16.9% studies that did not terminate before reaching any boundary, 6.3% showed evidence for H1 (BF_10_ > 3), 9.5% were inconclusive, and 0.6% showed evidence for H0 (BF_10_ < 1/3). In other words, if H1 is true, 89.2% of the studies will show evidence for H1, and only 0.7% will show evidence for H0.

It is important to note that our planned minimal sample size (i.e., 60 participants per condition) is already more than twice the size of previous studies in the field (Rigoni et al., 2011, 2012, 2013, 2015). An NHST for this minimal sample size would have 86% chance to detect the same effect size. Importantly, the simulation indicated that the average stopping point of the SBF is 73 participants in each group (a total of 146 participants), which enhances the chances to detect the effect to 91% in NHST. Yet, in this study we will not use NHST to analyse our data. This is because the second purpose of our investigation – namely, understanding the mechanism underlying the influence of disbelief in free will on cognitive control – requires a Bayesian analysis as it involves potential null effects. For this purpose, the simulation indicated that for *Cohen's d* = 0 (i.e., H0 is true), 58.2% of the studies terminated in strong support to H0 (i.e., BF_10_ < 1/10), and only 0.9% of the studies terminated in strong support for H1 (i.e., BF_10_ > 10). Out of the remaining 40.9% studies that did not terminate before reaching any boundary, 27.7% showed evidence for H0 (BF_10_ < 1/3), 12.7% were inconclusive, and only 0.5% showed evidence for H1 (BF_10_ > 3). In other words, if H0 is true, 85.9% of the studies would show evidence for H0, and only 1.4% would show evidence for H1.

We planned to run the first analysis only after we collected at least 60 participants in each group after exclusions (see Participants Exclusion section for exclusion specification). This meant that if due to exclusions participants’ number in a group drops below 60, we planned to continue running the study for another day before analysing. To reach at least 60 participants in each group, we ended up conducting three runs. We then continued with data collection, because we planned that if we do not reach either the 10 or the 1/10 boundary in all analyses, we planned to continue data collection and analyse it after each run until we reach a boundary (or n.max) for all analyses. We did not meet the boundaries after the first analysis (Runs 1 to 3) and conducted three more runs after that (Runs 4 to 6). We did not meet the boundaries after any of the runs, including the last, which lead to n.max (i.e., 100 participants per between‐subjects condition).^2^

### Procedure

The experiment started with an explanation and illustration of the switching task and a practice block of 64 trials. As in online studies participants may be less attentive, we let participants repeat the practice block until they met a minimum accuracy threshold of 75% accurate responses.^3^ If participants met the minimum accuracy threshold of 75% accurate responses, they continued with the main part of the experiment. If participants did not reach the threshold, they were informed that they made too many errors. Afterwards, they had to read the instructions again and then conduct another practice block. Participants could go through no more than three practice blocks. If participant were unable to reach the minimum threshold of 75% accuracy within the third block, they were directed to the end of the experiment and were not allowed to further participate in the study.

After the practice block(s), we manipulated participants' dis/belief in free will. The program assigned participants alternately to either the anti‐ or pro‐free will condition. Afterwards, participants performed seven experimental switching blocks of 64 trials each, rendering a total of 448 experimental trials. After completing the switching task, participants completed different questionnaires and indicated demographic information (study materials are available in OSF ‐ https://osf.io/7czr3/?view_only=f0e3c9db44594e578649bacaa1aa9634). The complete session took approximately 45 min (*M* = 43.6, *SD* = 10.8).

#### Free will manipulations

In order to manipulate belief in free will, we applied the belief in free will manipulation validated by Seto and Hicks (2016). We randomly assigned participants to read a brief description that promoted either an anti‐ or pro‐belief in free will. Participants in the pro‐free will belief condition read the following text:

Free will is defined as the ability to make our own choices and to determine our own outcomes. Most people believe in free will, and recent research supports this belief. For instance, even though some people still believe that their actions are greatly determined by outside influences (e.g., social pressures), behavioral economists and psychologists have published studies showing that most of our behavior is determined by personal choices.

Participants in the anti‐free will belief condition read the following text:

Free will is defined as the ability to make our own choices and to determine our own outcomes. Most people do not believe our behavior is completely determined by free will, and recent research supports this belief. For instance, many people believe that their actions are often determined by outside influences (e.g., social pressures). In fact, behavioral economists and psychologists have even published studies showing that most of our behavior is determined by situational factors.

After reading this short paragraph, participants were presented with the following 10 statements used in previous research to induce free will beliefs (Alquist et al., 2013):

1. I demonstrate my free will every day when I make decisions.
2. I take personal pride in good decisions I have made in the past because I know that, at the time, I had the freedom to and could have made a bad decision.
3. I am able to override the genetic and environmental factors that sometimes influence my behaviour.
4. Avoiding temptation requires that I exert my free will.
5. Ultimately people cannot blame their own actions on anything other than themselves.
6. I have free will to control my actions and ultimately to control my destiny in life.
7. People are responsible for their behaviours because they have free will to control their actions.
8. Our actions and thoughts are not simply the result of prior experiences.
9. By exerting their free will, people can and do overcome the negative effects of a dysfunctional environment.
10. Given that I have had personal experiences that science cannot explain, I also know that I have free will even if science cannot explain it.

We asked participants to think about why these statements are true (false) based on their own experiences. Moreover, participants in the anti‐free will belief condition then selected two to three statements that have proven especially false in their life. Conversely, participants in the pro‐free will belief condition selected statements that have proven especially true in their life. Finally, we asked participants to describe how each chosen statement is true (false) based on their own experiences and ‘think about specific examples from [their] life by providing as much detail as possible’.

#### Switching task

**FIGURE 1:**
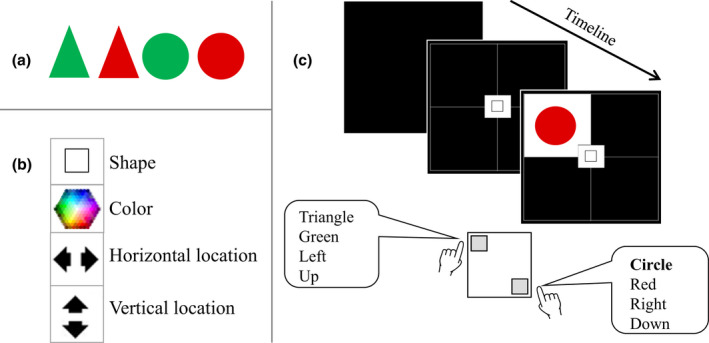
a) Stimuli. b) Task cues. c) Example of a trial and of cue–target pairs and response key arrangements. Stimuli and cues will be identical to those used in Katzir, Ori, Hsieh, et al., (2015b), Experiment 1A

We adjusted Katzir, Ori, Hsieh, et al.’s (2015b) task reported in Experiment 1A. In this task, participants switch classification between different S‐R sets. In line with previous research (e.g., Hsieh et al., 2012; Katzir et al., 2018; Katzir, Ori, Hsieh, et al., 2015b; Meiran et al., 2010, 2011), we let participants switch between four S‐R sets. This task is suitable to examine both the BI effect (which requires switching classification between at least three S‐R sets), and the CRS effect (which, due to the exclusions elaborated on below, is better estimated when switching between four tasks, as it leaves more trials for the final analysis than when switching between three tasks). The stimuli consist of an object presented inside a 2 × 2 grid subtending a visual angle of approximately 13.10 (width) × 13.10 (height) degrees. The object was a red or a green triangle (2.38° × 2.68°) or circle (diameter 2.38°), (See Figure 1a).

We instructed participants to classify stimuli, as fast and as accurate as they can, according to different dimensions of the stimuli: shape (circle, triangle), colour (red, green), vertical position (up, down), and horizontal position (left, right). The dimensions were indicated by task‐related icons that served as task cues (cf. Figure 1b). Task cues were presented in the centre of the grid, while the stimulus was presented in the grid's quadrants (Figure 1c). Responses for all tasks were mapped to the same two keys. The response key arrangement was counterbalanced between participants. Half of the participants used an upper‐left (‘E’) and lower‐right (‘N’) keys to indicate up/left or down/right, as Figure 1c shows, while the other half used upper‐right (‘U’) and lower‐left (‘C’) keys to indicate up/right or down/left (these keys were selected because they have the same location in QWERY, QWERTZ, and AZERTY keyboards). The object task's responses were also mapped to the same response keys in a counterbalanced order, resulting in 8 mapping conditions. We counterbalanced the 8 response mapping conditions in each experimental group (pro‐ vs. anti‐ free will).

A trial started with a response‐cue interval of 500 ms black screen presentation followed by the presentation of the task‐cue in the centre of the empty grid for 500 ms. The target stimulus was then added to the display, until a response was given (Figure 1c). The target stimulus was randomly chosen in each trial. A light grey ‘X’ was presented following errors. Task ordering was randomly determined under the constraint that all the trials involved a task switch (Katzir et al., 2018; Katzir, Ori, Eyal, et al., 2015a; Katzir, Ori, Hsieh, et al., 2015b; for justification see Meiran et al., 2010).

Participants performed either one, two, or three practice blocks (as explained above) and seven experimental blocks. Each block consists of 64 trials. The number of experimental blocks was selected to assure a minimal number of valid trials for the analysis. We did not want to extend the task further, assuming that the free will manipulation might be short lived. After each block, participants were able to take a break for as long as they want.

#### Control questions

##### Subjective fatigue

Given that most people believe in free will (Baumeister et al., 2009; Nahmias et al., 2005), it might be that participants in the anti‐free will group, as compared with participants in the pro‐free will group, get more fatigued by processing information that is contrary to their belief. To test whether fatigue, instead of belief in free will, accounts for the assumed effects, at the end of the switching task participants reported on 7‐point rating scales (1 *= not at all*; 7 = *very much*) how tired, bored, alert, and energetic they feel. The items were presented in a random order. To prepare data for analyses, we averaged all items (Cronbach's α = .79) to create the subjective fatigue score such that a high score indicates more subjective fatigue (for a similar procedure see Katzir et al., 2020).

##### Free will belief

After completing the switching task, participants filled in the free will subscale^4^ of the Free Will Inventory (Nadelhoffer et al., 2014). Participants rated their agreement on five statements on 7‐points rating scales (strongly disagree, disagree, somewhat disagree, neither agree nor disagree, somewhat agree, agree, strongly agree). The free will score was computed by averaging all the items of the free will subscale items (Cronbach's α = .87).

##### Locus of control

After completing the FWI, participants completed the 23‐items Locus of Control Scale (Rotter, 1966), in which participants are instructed to indicate, for each item, ‘which sentence you agree with by choosing either sentence (A) or sentence (B)’. For example, they must choose either ‘A ‐ Many of the unhappy things in people's lives are partly due to bad luck’ and ‘B ‐ People's misfortunes result from the mistakes they make’. To compute a locus of control score, we assigned a point to every internal locus statement chosen and then summed the points up, such that high values indicate a relatively strong internal, as compared to an external, locus of control. Cronbach's Alpha for the compound score was α = .80.

##### Suspicion

After answering the Locus of Control items, we asked participants ‘You completed several tasks and questionnaires in this Experiment. With regard to each of them, what do you think we expect to find? In other words, what were the hypotheses of the study?’

##### Demographic information

Before leaving the experiment, participants indicated their age, gender, and whether they are colour blind.

#### Data preparation

Before analysing the cognitive indices, we followed the procedure from Meiran et al. (2010) to prepare and exclude data (Hsieh et al., 2012; Katzir et al., 2018; Meiran et al., 2010). Specifically, the two consecutive trials that followed an error (as required in order to compute Backward Inhibition) and the first four trials in each block were removed. Also, we analysed RT only for correct trials and excluded responses with RT smaller than 100 ms or longer than 3000 ms. In addition, for each analysis, we computed the cell mean separately for each participant in each task as we elaborate below. We also excluded trials when the same stimulus is repeated in all dimensions (Hommel, 1998).

Because tasks were selected randomly (with a constraint that there are 100% switch), there may be under or over representations of some tasks in some conditions. Because spatial tasks (i.e., respond to the location of the object) are expected to be faster than object‐related tasks (i.e., respond to the colour or the shape of the object), we first computed the average for each participant in each task in each condition, and then averaged across tasks for each participant, in each condition. Computing the average for each task in each condition gives an equal weight for each task and thus prevents possible biases that under or over representation of tasks may have on the average in each condition (Katzir et al., 2018; Katzir, Ori, Eyal, et al., 2015a). In what follows, we specify how we determined the levels of each index variable.

##### Congruency effect

Because our design involved four tasks, the congruency effect has four levels that are determined by the number of competitor S‐R sets in a given trial. Specifically, in each trial there could either be 0, 1, 2, or 3 competitor S‐R sets. The congruency effect is a within variable that manifests as a linear trend and should be estimated using a linear contrast (Pinhas et al., 2012). For ease of analysis and interpretation, we computed a single index of the congruency effect by multiplying the four Number of Competitor Set conditions (0, 1, 2, or 3 competitors) by the weights of a linear contrast (−3, −1, 1, 3 for the conditions 0, 1, 2, 3, respectively). The sum of these products served as an index to represent the linear‐congruency effect for each participant (Katzir, Ori, Eyal, et al., 2015a).

##### Backward inhibition (BI)

Backward Inhibition (Mayr & Keele, 2000) is the cost (either in RT or in PE) seen when participants have to apply an S‐R set they have abandoned recently (e.g., applying the set A after having to abandon it in Trial N‐1, such as in the sequence A → B → **A** ) compared to trials in which this S‐R set was not recently abandoned (e.g., the sequence C → B → **A** ). An example for an AB **A** sequence in our paradigm is a sequence in which the relevant dimension for classification (i.e., the relevant S‐R set) in Trial N‐2 was colour (A), in Trial N‐1 it was shape (B), and in Trial N it was colour again (A). An example for a CB **A** sequence is a sequence in which the relevant dimension for classification in Trial N‐2 was vertical location (C), in Trial N‐1 it was shape (B), and in Trial N it was colour (A). The backward inhibition effect reflects the inhibition of a no longer relevant S‐R set that occurs in Trial N‐1, when one needs to abandon the relevant S‐R set from Trial N‐2. The relevant S‐R set from Trial N‐2 has to be inhibited in Trial N‐1 in order to improve execution of the relevant S‐R set in Trial N‐1(Koch et al., 2010; Mayr & Keele, 2000). This inhibition lingers to the next trial. If this inhibited S‐R set becomes the relevant S‐R set in trial N (i.e., an AB **A** sequence), its execution would be impaired compared to executing the same set in a sequence where it was not inhibited in trial N‐1 (i.e., a CB **A** sequence) (Mayr & Keele, 2000). To capture the trace of inhibition that lingers from Trial N‐1 to Trial N, AB **A** trials (i.e., trials that involve a trace of inhibition from Trial N–1), are compared to CB **A** trials (i.e., trials that do not involve a trace of inhibition from Trial N–1). Therefore, to compute the BI index, we subtracted CB **A** trials from AB **A** trials. Based on probability calculations and a simulation (the simulation code is available in OSF ‐ https://osf.io/7czr3/?view_only=f0e3c9db44594e578649bacaa1aa9634), this should result in ~280 trials, on average, for each participant on the CB **A** condition and ~ 140 trials on the AB **A** condition.

##### Competitor rule suppression (CRS)

Competitor Rule Suppression (Hsieh et al., 2012; Katzir et al., 2018; Meiran et al., 2010) is a sequential effect that manifests as the cost (either in RT or in PE) seen when comparing trials with lingering suppression (i.e., CRS+ trials, in which a competitor S‐R rule from Trial N‐1 becomes the relevant S‐R rule in Trial N), to trials with no lingering suppression (i.e., CRS‐ trials, in which the relevant S‐R rule in Trial N was not a competitor S‐R rule in Trial N‐1). For example, consider that in our task, circle and green are mapped to Key 1 and triangle and red are mapped to Key 2. Consider, also, a Trial N in which participants have to respond to the colour of a green circle (i.e., the relevant S‐R set is colour, and the correct response is green). In CRS+ trials, this Trial N was preceded by Trial N‐1 in which participants had to respond to the shale of a green triangle (i.e., the ‘green’ S‐R rule was a competitor rule, because ‘green’ is mapped to Key 1 and ‘triangle’ is mapped to Key 2). In CRS‐ trials, the same Trial N was preceded by Trial N‐1 in which participants had to respond to the shape of a green circle (i.e., the ‘green’ S‐R rule was *not* a competitor rule, because both ‘circle’ and ‘green’ are mapped to Key 1). Importantly, only Trial Ns are analysed (in our example, a trial in which colour is the relevant dimension for classification, and the shape is green), but what differentiates CRS+ trials from CRS‐ trials is whether the irrelevant S‐R rule in Trial N‐1 (i.e., ‘green’) was a competitor S‐R rule or not. Research has shown that the Competitor Rule Suppression effect reflects processes aimed at reducing the interference coming from interfering S‐R rules in Trial N‐1(Katzir et al., 2018). Suppressing the interfering S‐R rule(s) (the ‘green’ rule in our example) in Trial N‐1 lingers to Trial N and results in cost when a competing S‐R rule in Trial N‐1 becomes the relevant S‐R rule in Trial N (i.e., CRS+ trials). Therefore, the Competitor Rule Suppression effect is an index for cognitive control (Hsieh et al., 2012; Katzir et al., 2018; Meiran et al., 2010).

On the practical level, the Competitor Rule Suppression effect is expected to manifest as a cost in CRS+ trials (i.e., trials that involve a trace of suppression from Trial N‐1 because the relevant S‐R rule in Trial N was the interfering S‐R rule in Trial N‐1) compared to CRS‐ trials (i.e., trials that do not involve a trace of suppression from Trial N‐1 because the relevant rule in Trial N was not the competitor rule in Trial N‐1, Meiran et al., 2010). Therefore, to compute the CRS index, we subtracted CRS‐ trials from CRS+ trials. A large CRS effect reflects high cognitive control.

Importantly, because this effect is derived by the S‐R rule, but not by the S‐R set,^5^ we analysed this effect only in response alternation (Katzir et al., 2018). To assure that the suppression effect in fact reflects suppression and no other processes, we applied some strict exclusion criteria (Katzir et al., 2018; Katzir, Ori, Hsieh, et al., 2015b). These criteria were applied to specific trials to determine whether they were entered to the analysis. Specifically, we equated CRS+ trials to CRS– trials with respect to the degree of interference in Trial N (namely, the number of competitor S‐R sets, Meiran et al., 2010; Sudevan & Taylor, 1987), the degree of interference in Trial N‐1(Botvinick et al., 2001), Competitor Rule Priming (i.e., competitor S‐R set priming, Katzir, Ori, Hsieh, et al., 2015b), and Competitor Remains Competitor (Katzir et al., 2018). We also excluded trials in which all of the dimensions of the previous stimulus repeat (Hommel, 1998). An elaborated description of these confounds and how they are controlled can be found in prior work (Katzir et al., 2018; Katzir, Ori, Hsieh, et al., 2015b). Controlling for all of the above mentioned confounds is expected to reduce the number of valid trials. Based on our experience with this paradigm, and a simulation we ran, this would result in ~43 trials, on average, for each participant on each condition of CRS.

#### Participants exclusion

In the six runs combined, a total of 709 participants started the experiment, of which 99 did not finish it, 4 did not give consent, 9 found a way around the restrictions and tried to take the study more than once, and 21 participants failed to reach the threshold of 75% correct trials in three practice blocks, leaving a total of 576 participants, of which 373 participants were excluded based on the following preregistered exclusions:

##### Offset delay

To assure the quality of the data, Paolacci and Chandler (2014) mention that low technical quality of participants' computers (and internet connections) can negatively impact reaction‐time‐based measures. Thus, we programmed our experiment with Labvanced (Finger et al., 2017). The advantage of Labvanced is that it measures offset delays resulting from bad internet connection or other equipment‐related problems. As recommended by Labvanced and in line with our preregistration, we excluded participants (*N* = 209) whose Labvanced mean offset delay measure was below 30 ms and/or their Labvanced SD offset delay measure was below 36 ms.^6^

##### Overall performance

As preregistered and based on the recommendation by Leys et al. (2013), we also excluded participants whose overall performance in the switching task (in RT or in PE) deviated in more than 2.5 MADs from their experimental group's (i.e., pro‐ or anti‐free will) Median. We first calculated the PE based on all the data. One participant had more than 40% errors and was excluded from the analyses before the MADs were calculated. For the RT‐MAD exclusion, we applied the same exclusion criteria that was applied in the analysis, namely we removed incorrect trials and excluded responses with RT smaller than 100 ms or longer than 3000 ms. (a total of 54 participants were excluded based on the MAD RT or PE criterion).

##### Sufficient number of trials

In line with our preregistration, we also excluded participants who had less than 20 trials in each condition. Importantly, as explained above, we first computed the average RT and PE in each condition in each task, and then averaged across tasks. We excluded participants if they did not have at least 5 data points in each task in each condition (105 participants were excluded based on this criterion).

Finally, three more participants had missing data due to a coding error. Because we preregistered that we will only use complete data sets, we excluded them from the analyses. Following these exclusions, we ended with 101 participants in the pro‐free will condition, and 103 participants in the anti‐free will condition.

## Preregistered analyses

Based on the recommendation put forward for Sequential Bayes Factor (SBF) analyses by Schönbrodt et al., (2017), we used the most recent available JASP statistical software (JASP team, 2018) to compute Bayesian statistics in all the analyses. We computed JSZ Bayes Factors (BF_10_) with 1 as Cauchy priors for *t*‐tests. This method is suitable for accumulating evidence in favour of H0 (Rouder et al., 2009). Interpretation of BF_10_ follow commonly accepted norms in which anecdotal (1 < BF_10_ < 3 for H1; 1 > BF_10_ > 1/3 for H0), substantial (3 < BF_10_ < 10; 1/3 > BF_10_ > 1/10), strong (10 < BF_10_ < 30; 1/10 > BF_10_ > 1/30), very strong (30 < BF_10_ < 100; 1/30 > BF_10_ > 1/100), or decisive (100 < BF_10_; 1/100 > BF_10_) evidence are inferred from BF_10_ (Jeffreys, 1961). We also included η_p_^2^ and Cohen's d as effect size estimate calculated with JASP.

### Outcome‐Neutral Criteria

We defined one outcome‐neutral criteria that needs to be met for the planned analyses. Specifically, we tested the presence of each index (TRCE, BI, CRS) by performing Bayesian one‐sample *t*‐test for each index separately for RT and PE. Because the anti‐free will manipulation is expected to reduce cognitive control, it may lead to a dilution of these effects, rendering them non‐significant. Thus, as preregistered, we applied the analysis in the pro‐free will belief group. In addition, we report (although not preregistered) the same analysis on the entire data set.

As Table 1 shows, all of the outcome‐neutral criteria were met for the main analyses. Specifically, the evidence for the effects was decisive for all of the RT effects. We also preregistered that we will explore the effects in errors (PE). Two out of the three outcome neutral criteria for the PE analyses were met. Specifically, the evidence for the linear‐congruency effect in errors was decisive, and it was strong for the competitor rule suppression effect in errors (CRS‐PE). There was strong evidence for a lack of backward inhibition effect in errors (BI‐PE). Because we pre‐registered that, we will examine the influence of free‐will only on indices that will emerge in this analysis as an effect substantially larger than zero (i.e., BF >3), we did not test the influence of the free will belief manipulation on BI‐PE.

**TABLE 1:** Outcome Neutral Criteria analyses

	BF₁₀	Evidence accumulation	*M*	*SD*
Entire sample				
Linear – cong. – RT	1.31*10^38^	H1: decisive	338.05	282.73
Linear – cong. – PE	1.00*10^32^	H1: decisive	.17	.17
BI – RT	1.91*10^8^	H1: decisive	25.64	53.12
BI – PE	0.045	H0: strong	−.0004	.02
CRS – RT	2.89*10^3^	H1: decisive	27.91	85.98
CRS – PE	1.05*10^2^	H1: decisive	.008	.03
Pro‐free will condition				
Linear – cong. – RT	1.57*10^18^	H1: decisive	342.77	289.18
Linear – cong. – PE	3.78*10^16^	H1: decisive	.19	.17
BI – RT	1.12*10^3^	H1: decisive	24.47	55.53
BI – PE	0.15	H0: substantial	.002	.02
CRS – RT	2.79*10^2^	H1: decisive	35.01	87.14
CRS – PE	5.34*10^1^	H1: strong	.01	.03

*Note:* Results from the Outcome Neutral Criteria analyses.

Abbreviations: RT = response time, PE = proportion of errors, Linear – cong. = Linear congruency effect, BI = backward inhibition, CRS = competitor rule suppression. BF₁₀ = Bayes Factor in favour of H1 over H0.

#### Manipulation check (not preregistered)

Although we did not preregister a manipulation check, we wanted to assure that the manipulation influenced belief in free will. We found that the manipulation decisively influenced participants' belief in free will as reflected in their self‐reports in the Free Will Inventory (FWI), BF_10_ = 1.18*10^4^, η_p_^2^ = .11, *d* = 0.70. Participants in the pro‐free will condition reported more belief in free will (*M* = 4.65, *SD* = 1.20) than participants in the anti‐free will condition (*M* = 3.79, *SD* = 1.23).

#### Suspicion

None of the participants correctly guessed the study hypothesis.

### Main analyses

#### Preregistered Primary analyses

In order to test the influence of the free will belief manipulation on each of the indices, we first ran five Bayesian independent‐sample *t*‐tests, according to the preregistered plan. For the Congruency Effect, we conducted two Bayesian independent‐sample *t*‐tests, one on the RT‐linear‐congruency index and one on PE‐linear‐congruency index, with belief in free will (anti‐free will vs. pro‐free will) as the grouping variable. We expected a larger congruency index in the anti‐free will belief group compared to the pro‐free will belief group. This effect should be obtained in RT. In addition, we explored whether it is also obtained in PE.

For Backward Inhibition, we conducted one Bayesian independent‐sample *t*‐test on the RT‐BI index, with belief in free will (anti‐free will vs. pro‐free will) as the grouping variable. According to the Control‐Prediction, we expected to accumulate evidence in favour of a smaller BI index effect in the anti‐free will belief group compared to the pro‐free will belief group (i.e., BF_10_ > 1). This effect should be obtained in RT. According to the Automaticity‐Prediction, we expected to accumulate evidence in favour of H0 indicating a null effect of the anti‐ (compared to the pro‐) free will manipulation on the BI index (BF_10_ < 1).

For Competitor Rule Suppression, we conducted two Bayesian independent‐sample *t*‐tests, one on the RT‐CRS index and one on PE‐CRS index, with belief in free will (anti‐free will vs. pro‐free will) as the grouping variable. According to the Control‐Prediction, we expected to accumulate evidence in favour of a smaller CRS index effect for RTs in the anti‐free will belief group as compared to the pro‐free will belief group (i.e., BF_10_ > 1). In addition, we ran the same analyses for PE. According to the Automaticity‐Prediction, we expected to accumulate evidence in favour of H0 indicating a null effect of the free will belief manipulation on the CRS index (BF_10_ < 1).

As Table 2 shows, all of the Bayesian independent‐sample *t*‐tests accumulated evidence in favour of the null hypothesis, namely, the free will manipulation did not influence any of the indices.

**TABLE 2:** Primary analyses

	BF₁₀	η_p_^2^	*d*	Evidence accumulation	*M*_pro_	*M*_anti_	*SD*_pro_	*SD*_anti_
Linear – cong. – RT	0.09	<.0001	−0.03	H0: strong	342.77	333.42	289.18	277.60
Linear – cong. – PE	0.04	.014	−0.23	H0: strong	.19	.16	.17	.15
BI – RT	0.09	<.0001	−0.04	H0: strong	24.47	26.79	55.53	5.87
BI – PE	–	–	–	–	.002	−.002	.02	.02
CRS – RT	0.37	.007	0.16	H0: anecdotal	35.01	2.94	87.14	84.66
CRS – PE	0.58	.011	0.21	H0: anecdotal	.01	.005	.03	.03

*Note:* Results from the primary analyses.

Abbreviations: RT = response time, PE = proportion of errors, Linear – cong. = Linear congruency effect, BI = backward inhibition, CRS = competitor rule suppression. BF₁₀ = Bayes Factor in favour of H1 over H0. The sign of Cohen's d indicates whether the means were in the predicted direction.

#### Preregistered secondary analyses

To further investigate the results, we ran preregistered Bayesian correlations between the composite score of the FWI's free will subscale and all indices in the pro‐free will belief group. The results are depicted in Table 3.

**TABLE 3:** Secondary analyses

	*r*	BF₁₀	Evidence accumulation
Linear – cong. – RT	.017	0.109	H0: substantial
Linear – cong. – PE	.033	0.098	H0: strong
BI ‐ RT	.043	0.181	H0: substantial
BI – PE	–	–	–
CRS – RT	−.063	0.081	H0: strong
CRS – PE	.073	0.245	H0: substantial

*Note:* Results from the correlation analyses between the FWI (free will inventory) and each of the indices.

Abbreviations: RT = response time, PE = proportion of errors, Linear – cong. = Linear congruency effect, BI = backward inhibition, CRS = competitor rule suppression. BF₁₀ = Bayes Factor in favour of H1 over H0.

#### Alternative explanations

##### Fatigue

To examine whether participants in the anti‐free will group felt more fatigued than those in the pro‐free will group, we conducted a preregistered Bayesian independent‐sample *t*‐test on the subjective fatigue score, with belief in free will (anti‐free will vs. pro‐free will) as the grouping variable. We expected to accumulate evidence in favour of H0 indicating a null effect of the anti‐ (compared to the pro‐) free will manipulation on subjective fatigue. As a more objective measure of fatigue, we also used the overall performance RT score, assuming that fatigue will lead to slower RTs. We thus conducted a Bayesian independent‐sample *t*‐test on the overall RT score, with belief in free will (anti‐free will vs. pro‐free will) as the grouping variable. Because Rigoni et al. (2012) did not find that an anti‐free will manipulation leads to slower RTs, we predicted that here, too, there will be no group differences.

As predicted, the manipulation did not influence participants' fatigue. There were substantial evidence favouring the null hypothesis for subjective self‐reports, BF_01_ = 3.72, and for overall RT performance, BF_01_ = 5.41.

##### Locus of control

To examine the extent to which the free will manipulation influenced control and/or automaticity independent of locus of control, we preregistered to run six separate multiple regression analyses on each of the six dependent measures (RT‐linear‐congruency index, PE‐linear‐congruency index, RT‐BI, PE‐BI, RT‐CRS, and PE‐CRS) as a dependent measure by statistically controlling for locus of control. We also preregistered that in each regression, manipulated free will (anti‐free will coded −0.5 and pro‐free will coded +0.5) will be one independent variable, and locus of control another independent variable. However, because we did not replicate previous findings (i.e., the belief in free will manipulation did not influence any of the cognitive indices), and because locus of control did not correlate with any of the indices (all BF_10_ < 0.74, not preregistered), nor was it impacted by the manipulation (BF_10_ = 0.17, not preregistered), it was already clear before running the preregistered analyses that they will support the null model. Nevertheless, for reasons of transparency and completeness, we report the analyses in the SOM.

## Exploratory and not preregistered analyses

To further explore the data, we first examined whether the preregistered analyses yield different results when we do not exclude any of the participants. We prepared the data for analysis, but, because some data sets were either incomplete, or participants did not have enough trials in one condition for the analysis, the different analyses involve different Ns. Readers are referred to the SOM for the analyses and the number of participants in each analysis (available in the tables).

When no participants were excluded from the analyses, the results remained essentially similar. The outcome neutral criteria yielded decisive evidence for all of the cognitive indices apart from errors in backward inhibition, that yielded very strong evidence for the null hypothesis. The manipulation check still had substantial influence, BF_10_ = 9.42*10^14^, η_p_^2^ = .12, *d* = .75 (in the pro‐free will condition, *M* = 4.72 and *SD* = 1.21, *n* = 309; in the anti‐free will condition, *M* = 3.83 and *SD* = 1.20, *n* = 272). The main analyses mostly yielded strong evidence in support of the null hypothesis. The secondary analysis indicated that self‐report measure for free‐will belief (i.e., FWI score) and any of the cognitive indices did not correlate (There were either substantial or strong evidence supporting the null hypothesis in all of the indices, apart from the correlation with errors in the competitor rule suppression (CRS) effect, in which the evidence was indecisive, BF_10_ = 1.02). There were still evidence favouring the null hypothesis for subjective fatigue self‐reports, BF_01_ = 13.30, and for overall RT performance, BF_01_ = 14.98, which served as a more objective measure of fatigue.

Interestingly, when the entire sample was analysed, we found a decisive impact of the free will belief manipulation on locus of control self‐reports, BF_10_ = 428.19 (in the pro‐free will condition, *M* = 9.60 and *SD* = 4.41, *n* = 311; in the anti‐free will condition, *M* = 8.12 and *SD* = 3.94, *n* = 273). Yet, even though Locus of control correlated with the free will self‐report scores (FWI scores) both in the sample after the preregistered exclusions (*r* = .38, BF_10_ = 7.14*10^5^, *n* = 204), and the sample with no exclusions (*r* = .42, BF_10_ = 8.62*10^22^, *n* = 581), the preregistered regression analyses with manipulated free will (anti‐free will coded −0.5 and pro‐free will coded +0.5) and locus of control as independent variables had no impact on any of the cognitive indices. For correlational analyses between locus of control, FWI, and the cognitive indices, see SOM.

## DISCUSSION

The goal of the current investigation was to shed light onto the exact process that underlie the previously documented connection between disbelief in free will and self‐control reduction. Specifically, we sought to conceptually replicate the previously reported influence of reducing belief in free will on cognitive control mechanisms (Rigoni et al., 2011, 2012, 2013, 2015), and to answer the question of whether it makes people less controlled or whether it enhances their reliance on automatic impulses. To do that, we used a task switching paradigm, which enables the extraction of various cognitive indices that may reflect either pure cognitive control (backward inhibition and competitor rule suppression) or an index that reflects both control and automaticity (the linear congruency effect). Our experiment did not conceptually replicate previous findings by Rigoni and colleagues. Although the experimental manipulation influenced participants' belief in free will, we did not find evidence for an effect on any of the cognitive indices extracted from a task switching paradigm. In fact, the main analyses provided evidence in favour of the null hypothesis, suggesting that manipulating belief in free will does neither influence automaticity nor does it influence cognitive control.

### Potential reasons for the null effect

There might be different reasons, why we did not find the predicted effects. First, previous studies that documented an effect of disbelief in free will on cognitive control were conducted in the laboratory. The current investigation was conducted online. Thus, one could argue that the failed replication is based on low‐quality data. However, in this respect it is important to highlight that we took great care to assure high quality of the data by restricting participants based on qualifications, by introducing a performance contingent reward, by measuring the quality of the data from a technical aspect, and by introducing participants with a novel task in a setup in which they are not likely to realize the study hypotheses (i.e., extracting unfamiliar cognitive indices; Paolacci & Chandler, 2014). Thus, we are convinced that data quality is not likely to be the reason for the failed replication. This assertion is further supported by our findings. Our investigation yielded five out of the six outcome‐neutral criteria – namely, we obtained strong to decisive evidence to five out of the six cognitive indices, and importantly, they were decisive in all three RT measures, which were at the focus of our investigation – thus indicating that the data had a high quality in detecting cognitive effects, yet it failed in detecting an influence of a disbelief in free will manipulation on these indices.

Second, another reason for the failed replication could be based on the free will belief manipulation we used. However, we detect with a BF_10_ = 1.18*10^4^ a very strong effect on the manipulation check (i.e., participants' belief in free will). This finding rules out the possibility that we did not replicate previous findings because the manipulation had no impact on participants' beliefs. Moreover, this result is in line with a current meta‐analysis (Genschow et al., in press) showing that in comparison to other validated experimental free will belief manipulations, the manipulation we chose here produces the strongest effects.

Third, one may argue that our sample of 204 participants is too small to detect the proposed effect. However, the Bayesian analyses support the null effect. It is, thus, rather unlikely that we would find the predicted effect with a larger sample. Even if one would detect a significant effect with a larger sample, the effect at hand would be very small and, thus, rather neglectable.

Fourth, it might be that belief in free will is not related with cognitive control and the original findings were false positives. Indeed, our failed conceptual replication joins a recent direct failed replication of the influence of manipulating belief in free will on the post‐error slowing effect (Eben et al., 2020), further casting doubt on the influence diminishing belief in free will may have on cognitive control, and more broadly, on self‐control.

Fifth, it could be that free will beliefs are related with cognitive control, but the applied free will belief manipulation was not suited for this purpose. Although we selected the manipulation that produces the strongest effects, a recent meta‐analysis (Genschow et al., in press) indicates that typical free will belief manipulations produce rather unspecific effects in the sense that they not only influence belief in free will, but also other beliefs such as the belief in dualism. In this research, we tested whether our free will belief manipulation influences other factors and found that it does influence belief in free will, but not fatigue. However, depending on how we exclude participants, we found an effect of the manipulation on Locus of Control. This indicates that the manipulation may indeed not produce very specific effects. In addition, it might well be that the manipulation influenced other psychological variables we did not assess. These variables might then have counteracted the influence of the manipulation on cognitive control.

Sixth, one could argue that the method we used to measure cognitive control is not suited to detect the predicted effect. However, we believe that the task we used is suitable for several reasons. First, there are strong arguments and empirical support to the notion that the backward inhibition and the competitor rule suppression effects are relatively pure indices for cognitive control (Hsieh et al., 2012; Katzir et al., 2018; Koch et al., 2010). Thus, the constellation of comparing an impact on them in combination with an impact on the congruency effect, a well‐known conflict measure (Kiesel et al., 2007; Meiran & Kessler, 2008),which reflects both control and automaticity, is a valid approach that renders dissociative results (Katzir, Ori, Eyal, et al., 2015a). Moreover, the cognitive indices that we extracted from a task switching paradigm had produced reliable differences between conditions that are known to influence cognitive control, such as the congruency effect in ADHD (e.g., Cepeda et al., 2000; King et al., 2007) and old age (e.g., Eppinger et al., 2007; Meiran et al., 2001). Importantly, cognitive indices in task switching have showed sensitivity to different manipulations, such as various mindfulness training (Greenberg et al., 2013, 2017), and affective manipulations (Jiang & Xu, 2014; Katzir, Ori, Eyal, et al., 2015a). Thus, despite the great promise that the task switching paradigm holds for understanding cognitive processes, it is possible that the impact of a belief in free will manipulation is only seen when one applies the exact same methodology used by Rigoni et al.

### Implications and further directions

Taken together, our experiment indicates that manipulating individuals' belief in free will does not affect cognitive control in a substantial manner. To further investigate the link between free will beliefs and cognitive control, future research could go in different directions. On the one hand, future research should develop more specific belief manipulations that affect only belief in free will, but not other variables. Using such manipulations may produce the effects we proposed. On the other hand, one should directly replicate the original findings on cognitive control (Rigoni et al., 2011, 2012, 2013, 2015) with the exact same manipulations and measures to test whether our failed replication is due to the change in the paradigm. Given that these two approaches do not produce the assumed effects, one could apply a correlational approach in which individuals' belief in free will is measured and then correlated with cognitive control abilities. Although we do not find an effect on a correlational level in our experiment, it might well be that without manipulating participants' belief in free will, the proposed relationship can be detected on an interindividual level. Indeed, several studies found on a correlational level robust links between belief in free will and several social‐cognitive outcomes, such as retributive punishment (Genschow et al., 2017; Martin et al., 2017), intentional attributions (Genschow et al., 2017, 2019; Genschow & Lange, 2022), and just world beliefs, religious worldviews, as well as a conservative world view (Carey & Paulhus, 2013; Genschow & Vehlow, 2021) – to name just a few examples. These findings suggests that on an interindividual level, belief in free will could well be connected to relevant social‐cognitive parameters.

Another interesting observation comes from a recent study conducted by Zhao et al. (2021). The authors suggest that the causal association between self‐control and belief in free will may be reversed to what we examined in the current investigation. Specifically, the authors found that self‐control success or failure influences free will beliefs. Given the correlational associations found between free will belief and other psychological factors, it would be interesting to further examine the role one's own self‐control success and failure plays in shaping these societal relevant beliefs and behaviours.

Besides these insights, our findings have also important implications for current philosophical debates. Since cognitive neuroscientists put forward that humans' perception of free will is nothing more than an illusion (e.g., Crick, 1994; Harris, 2012; Wegner, 2002), anti‐free will viewpoints became en vogue not only in academia (e.g., Zeki et al., 2004), but also in popular press (e.g., Chivers, 2010; Griffin, 2016; Racine et al., 2017; Wolfe, 1997). When research in psychology found that confronting individuals with anti‐free will messages influences fundamental behaviours, cognition, and attitudes, the question arose of whether the media should distribute anti‐free will viewpoints. On the one hand, some philosophers argue that publishing anti‐free will messages would have negative consequences, because free will forms the basis for moral behaviour (e.g., Smilansky, 2003, 2005). On the other hand, some philosophers argue that reducing people's free will belief might also have positive effects, as it could lead to abandoning retribution‐based morality and illusory beliefs in a just world (Caruso, 2014; Nadelhoffer, 2011; Zeki et al., 2004). The present research adds to this debate by suggesting that confronting individuals with anti‐free will viewpoints might have, at least in the case of cognitive control, not as strong consequences as it has been previously assumed.

## AUTHOR CONTRIBUTIONS

**Maayan Katzir:** Conceptualization; formal analysis; investigation; methodology; project administration; software; writing – original draft; writing – review and editing. **Oliver Genschow:** Conceptualization; funding acquisition; methodology; writing – original draft; writing – review and editing.

## CONFLICTS OF INTEREST

All authors declare no conflict of interest.

### OPEN RESEARCH BADGES

This article has been awarded Open Data, Open Materials, Preregistered Research Designs Badges. All materials and data are publicly accessible via the Open Science Framework at https://osf.io/7czr3/?view_only=f0e3c9db44594e578649bacaa1aa9634.

## Data Availability

Data and materials are available at OSF: https://osf.io/7czr3/?view_only=f0e3c9db44594e578649bacaa1aa9634

## Footnotes

^1^
We chose 1 as Cauchy priors because this is the recommended procedure (Rouder et al., 2009; Schönbrodt et al., 2017). Note that using Cauchy priors lower than 1 would “make it rather difficult to reach the H0 boundary with a reasonable sample size” (Schönbrodt et al., 2017, p. 332), an outcome we wished to avoid.

^2^
Of note, instead of running the study at the university, as originally planned, we ended up running the study online. The original online sampling plan was approved by the editor and was preregistered (https://osf.io/8hx3w). The plan was modified after the second run due to the high unexpected rate of exclusions (https://osf.io/kqnez) and again before the last run, when we were close to n.max and needed less participants than planned (https://osf.io/g7dz6). How many participants were collected and excluded in each run is reported in the SOM.

^3^
This deviates from the original plan in Stage 1, but it was approved by the editor and was preregistered (https://osf.io/8hx3w).

^4^
We chose to only focus on the free will subscale because determinism (Nahmias et al., 2005, 2007, 2014; Nichols & Knobe, 2007) and dualism (Monroe et al., 2014; Monroe & Malle, 2010) seem to be distinct constructs.

^5^
The original CRS effect referred to repetitions of S‐R sets from Trial N‐1 to Trial N, not to repetitions of S‐R rules (Meiran et al., 2010). In a recent work, we showed that the CRS effect is only present when the S‐R rule repeats in Trial N, and not when the other S‐R rule that belongs to the same S‐R set appears in Trial N. Therefore, in this work we use the term CRS although we will only analyse trials with response alternations, as recommended in Katzir et al. (2018), in order to get a better estimation of the size of the suppression effect.

^6^
This deviates from the original plan in Stage 1, because due to the COVID‐19 outbreak, we had to switch from a lab to an online experiment. The adapted exclusion criteria were approved by the editor and was preregistered before running the experiment (see https://osf.io/8hx3w).
