# Supplementary material for: Automatic or controlled: How does disbelief in free will influence cognitive functioning?
Source: Br J Psychol. 2022 Jun 15;113(4):1121–42. doi: 10.1111/bjop.12578 (PMC9796308; doi:10.1111/bjop.12578)
Supplement: Supplementary file 1 [file BJOP-113-1121-s001.docx]

**Table of contents**

| Simulation code……………………………………………………………………... | 1 |
| --- | --- |
| Description of the sampling procedure……………………………………………... | 2 |
| Errors that were discovered at later stages…………………………………….. | 3 |
| Results from all analyses……………………………………………………………. | 4 |
| Analysis 1………………………………………………………………………... | 4 |
| Analysis 2………………………………………………………………………... | 6 |
| Analysis 3………………………………………………………………………... | 9 |
| Analysis 4………………………………………………………………………... | 11 |
| No Exclusions Analysis…………………………………………………………. | 21 |

**Simulation Code**

The simulation was run using the BFDA R-package:

sim.H1 <- BFDA.sim(expected.ES=0.5, type="t.between", prior=list("Cauchy",list(prior.location=0, prior.scale=1)), n.min=60, n.max=100, alternative="greater", boundary=c(1/10, 10), B=10,000, verbose=TRUE, cores=4, stepsize = NA)

sim.H0 <- BFDA.sim(expected.ES=0, type="t.between", prior=list("Cauchy", list(prior.location=0, prior.scale=1)), n.min=60, n.max=100, alternative="greater", boundary=c(1/10, 10), B=10,000, verbose=TRUE, cores=4, stepsize = NA)

BFDA.analyze(sim. H1, design="sequential", n.min=60, n.max=100, boundary=c(1/10, 10))

BFDA.analyze(sim.H0, design="sequential", n.min=60, n.max=100, boundary=c(1/10, 10))

plot(sim.H1, n.min=60, n.max=100, boundary=c(1/10, 10))

plot(sim.H0, n.min=60, n.max=100, boundary=c(1/10, 10), forH1 = FALSE)

**Description of the sampling procedure**

In the first run we opened 144 places and thought we ended up with 44 participants^[[1]](#footnote-1)^ (24 in the pro-free will condition, and 20 in the anti-free will condition); in the second run we opened 152 places and ended up with a total of 98 (50 in the pro-free will condition, and 48 in the anti-free will condition); and in the third run we opened 90 places, and ended with a total of 137 participants (65 in the pro-free will condition, and 72 in the anti-free will condition). We then ran the first analysis and did not reach the boundaries. We then continued with data collection, because we planned that if we do not reach either the 10 or the 1/10 boundary in all analyses, we will continue data collection and analyze after each run until we reach a boundary (or n.max) for all analyses. We conducted three more runs after the first analysis, until we reached n.max. In Run 4, we opened 80 places, and ended up with 161 participants (77 in the pro-free will condition, and 84 in the anti-free will condition); In Run 5, we opened 80 places, and ended up with 191 participants (92 in the pro-free will condition, and 99 in the anti-free will condition); In Run 6, we opened 40 places (30 in the pro-free will condition, and 10 in the anti-free will condition), and ended up with 209 participants (105 in the pro-free will condition, and 104 in the anti-free will condition). Of note, the original sampling plan that was pre-registered (<https://osf.io/8hx3w>) was modified after the second run due to the high unexpected rate of exclusions (<https://osf.io/kqnez>) and again before the last run, when we were close to n.max and needed less participants than planned (<https://osf.io/g7dz6>).

**Errors that were discovered at later stages**

After we ended running the study, we realized that there was a code error. The exclusions based on MAD was done on the entire sample, and not by group as was preregistered. This led to the exclusion of one participant from Run 1 and one participant from Run 6, but would have not dramatically change data collection and did not dramatically change the results.

Also, after Run 5, I discovered that I did not force response in the last FWI item. I fixed it before Run 6. This led to losing a total of 3 participants, 2 in Run 4, and one in Run 5).

All of the data reported here and in the paper are on the sample that after discovering the errors (i.e., the data from the previous analyses were re-analyzed).

|  | Before discovering the code/programming errors | | | | After discovering the error | | |  |
| --- | --- | --- | --- | --- | --- | --- | --- | --- |
|  | pro | anti | total | Accumul | pro | anti | total | Accumul |
| Run 1 | 24 | 20 | 44 | **44** | 23 | 20 | 43 | **43** |
| Run 2 | 26 | 28 | 54 | **98** | 26 | 28 | 54 | **97** |
| Run 3 | 15 | 24 | 39 | **137** | 15 | 24 | 39 | **136** |
| Run 4 | 12 | 13 | 25 | **162** | 11 | 12 | 23 | **159** |
| Run 5 | 16 | 15 | 31 | **193** | 15 | 15 | 30 | **189** |
| Run 6 | 12 | 4 | 16 | **209** | 11 | 4 | 15 | **204** |
| **Total** | **105** | **104** | **209** |  | **101** | **103** | **204** |  |

**Results From all analyses**

**Names of the variables in the JASP files:**

| TRCE_mrt_effect | Linear congruency effect - RT |
| --- | --- |
| TRCE_effect_merr | Linear congruency effect - PE |
| BI_mrt_effect | Backward inhibition - RT |
| BI_effect_merr | Backward inhibition - PE |
| crs_mrt_effect | Competitor rule suppression - RT |
| crs_effect_merr | Competitor rule suppression - PE |

**Analysis 1**

**Outcome neutral – based on the pro free will group**

## Bayesian One Sample T-Test

| **Bayesian One Sample T-Test** | | | | | | | | | | | | | | |
| --- | --- | --- | --- | --- | --- | --- | --- | --- | --- | --- | --- | --- | --- | --- |
|  | | | | | | | | | **BF₊₀** | | | | **error %** | |
| TRCE_mrt_effect | | | | | | | |  | 9.275e+11 | | |  | NaN | ᵃ |
| TRCE_effect_merr | | | | | | | |  | 1.670e+9 | | |  | NaN | ᵃ |
| BI_mrt_effect | | | | | | | |  | 23.741 | | |  | ~ 8.954e-5 |  |
| BI_effect_merr | | | | | | | |  | 0.185 | | |  | ~ 4.111e-5 |  |
| crs_mrt_effect | | | | | | | |  | 159.151 | | |  | ~ 1.325e-5 |  |
| crs_effect_merr | | | | | | | |  | 3.925 | | |  | ~ 2.910e-5 |  |
|  | | | | | | | | | | | | | | |
| Note.  For all tests, the alternative hypothesis specifies that the population mean is greater than 0. | | | | | | | | | | | | | | |
| ᵃ t-value is large. A Savage-Dickey approximation was used to compute the Bayes factor but no error estimate can be given. | | | | | | | | | | | | | | |
| **Descriptives** | | | | | | | | | | | | | |  |
|  | | | | | | | | | | **95% Credible Interval** | | | |  |
|  | | **N** | | **Mean** | | **SD** | | **SE** | | **Lower** | | **Upper** | |  |
| TRCE_mrt_effect |  | 64 |  | 301.161 |  | 242.979 |  | 30.372 |  | 240.466 |  | 361.855 |  |  |
| TRCE_effect_merr |  | 64 |  | 0.195 |  | 0.188 |  | 0.024 |  | 0.148 |  | 0.242 |  |  |
| BI_mrt_effect |  | 64 |  | 19.631 |  | 48.427 |  | 6.053 |  | 7.534 |  | 31.727 |  |  |
| BI_effect_merr |  | 64 |  | 0.002 |  | 0.023 |  | 0.003 |  | -0.004 |  | 0.008 |  |  |
| crs_mrt_effect |  | 64 |  | 40.568 |  | 83.354 |  | 10.419 |  | 19.747 |  | 61.389 |  |  |
| crs_effect_merr |  | 64 |  | 0.010 |  | 0.031 |  | 0.004 |  | 0.002 |  | 0.018 |  |  |
|  | | | | | | | | | | | | | |  |

Outcome neutral criteria were met in all criteria except for the BI in errors. Because we were mostly interested in RT, we ran the analyses.

Both hypotheses were directional but in opposite directions:

**For the TRCE**, we predicted that the TRCE will be reduced in the pro- compared to the anti- free will condition. This pattern was expected both in the control-prediction and in the automaticity prediction:

## Bayesian Independent Samples T-Test: TRCE

| **Bayesian Independent Samples T-Test** | | | | | | | | | | | | | | | | |
| --- | --- | --- | --- | --- | --- | --- | --- | --- | --- | --- | --- | --- | --- | --- | --- | --- |
|  | | | | | | | | | | | **BF₀₊** | | | | **error %** | |
| TRCE_mrt_effect | | | | | | | | | |  | 1.803 | | |  | ~ 3.000e-4 |  |
| TRCE_effect_merr | | | | | | | | | |  | 16.458 | | |  | ~ 0.008 |  |
|  | | | | | | | | | | | | | | | | |
| Note.  For all tests, the alternative hypothesis specifies that the location of group anti is greater than the location of group pro . | | | | | | | | | | | | | | | | |
| **Descriptives** | | | | | | | | | | | | | | | |  |
|  | | | | | | | | | | | | **95% Credible Interval** | | | |  |
|  | | **Group** | | **N** | | **Mean** | | **SD** | | **SE** | | **Lower** | | **Upper** | |  |
| TRCE_mrt_effect |  | anti |  | 72 |  | 360.302 |  | 273.192 |  | 32.196 |  | 296.105 |  | 424.499 |  |  |
|  |  | pro |  | 64 |  | 301.161 |  | 242.979 |  | 30.372 |  | 240.466 |  | 361.855 |  |  |
| TRCE_effect_merr |  | anti |  | 72 |  | 0.157 |  | 0.148 |  | 0.017 |  | 0.122 |  | 0.192 |  |  |
|  |  | pro |  | 64 |  | 0.195 |  | 0.188 |  | 0.024 |  | 0.148 |  | 0.242 |  |  |
|  | | | | | | | | | | | | | | | |  |

**For the Bi & CRS effects**, we had two predictions. According to the control prediction, we predicted that the effects will be enhanced in the pro- compared to the anti- free will condition. According to the automaticity prediction, we predicted that it will not differ. We thus tested the directional hypothesis for both predictions:

## Bayesian Independent Samples T-Test: BI & CRS

| **Bayesian Independent Samples T-Test** | | | | | | | | | | | | | | | | |
| --- | --- | --- | --- | --- | --- | --- | --- | --- | --- | --- | --- | --- | --- | --- | --- | --- |
|  | | | | | | | | | | | **BF₀₊** | | | | **error %** | |
| BI_mrt_effect | | | | | | | | | |  | 2.390 | | |  | ~ 1.465e-4 |  |
| BI_effect_merr | | | | | | | | | |  | 12.812 | | |  | ~ 0.005 |  |
| crs_mrt_effect | | | | | | | | | |  | 15.262 | | |  | ~ 0.007 |  |
| crs_effect_merr | | | | | | | | | |  | 15.898 | | |  | ~ 0.008 |  |
|  | | | | | | | | | | | | | | | | |
| Note.  For all tests, the alternative hypothesis specifies that the location of group anti is greater than the location of group pro . | | | | | | | | | | | | | | | | |
| **Descriptives** | | | | | | | | | | | | | | | |  |
|  | | | | | | | | | | | | **95% Credible Interval** | | | |  |
|  | | **Group** | | **N** | | **Mean** | | **SD** | | **SE** | | **Lower** | | **Upper** | |  |
| BI_mrt_effect |  | anti |  | 72 |  | 29.263 |  | 51.333 |  | 6.050 |  | 17.200 |  | 41.325 |  |  |
|  |  | pro |  | 64 |  | 19.631 |  | 48.427 |  | 6.053 |  | 7.534 |  | 31.727 |  |  |
| BI_effect_merr |  | anti |  | 72 |  | -0.001 |  | 0.020 |  | 0.002 |  | -0.006 |  | 0.004 |  |  |
|  |  | pro |  | 64 |  | 0.002 |  | 0.023 |  | 0.003 |  | -0.004 |  | 0.008 |  |  |
| crs_mrt_effect |  | anti |  | 72 |  | 23.629 |  | 86.599 |  | 10.206 |  | 3.279 |  | 43.979 |  |  |
|  |  | pro |  | 64 |  | 40.568 |  | 83.354 |  | 10.419 |  | 19.747 |  | 61.389 |  |  |
| crs_effect_merr |  | anti |  | 72 |  | 0.004 |  | 0.027 |  | 0.003 |  | -0.003 |  | 0.010 |  |  |
|  |  | pro |  | 64 |  | 0.010 |  | 0.031 |  | 0.004 |  | 0.002 |  | 0.018 |  |  |
|  | | | | | | | | | | | | | | | |  |

We did not reach boundaries, and therefore we continued with data collection.

**Analysis 2**

**Outcome neutral – based on the pro free will group**

## Bayesian One Sample T-Test

| **Bayesian One Sample T-Test** | | | | | | | | | | | | | | |
| --- | --- | --- | --- | --- | --- | --- | --- | --- | --- | --- | --- | --- | --- | --- |
|  | | | | | | | | | **BF₊₀** | | | | **error %** | |
| TRCE_mrt_effect | | | | | | | |  | 6.994e+13 | | |  | NaN | ᵃ |
| TRCE_effect_merr | | | | | | | |  | 1.415e+11 | | |  | NaN | ᵃ |
| BI_mrt_effect | | | | | | | |  | 263.740 | | |  | ~ 3.465e-5 |  |
| BI_effect_merr | | | | | | | |  | 0.249 | | |  | ~ 1.427e-5 |  |
| crs_mrt_effect | | | | | | | |  | 278.083 | | |  | ~ 3.878e-5 |  |
| crs_effect_merr | | | | | | | |  | 23.506 | | |  | ~ 2.854e-4 |  |
|  | | | | | | | | | | | | | | |
| Note.  For all tests, the alternative hypothesis specifies that the population mean is greater than 0. | | | | | | | | | | | | | | |
| ᵃ t-value is large. A Savage-Dickey approximation was used to compute the Bayes factor but no error estimate can be given. | | | | | | | | | | | | | | |
| **Descriptives** | | | | | | | | | | | | | |  |
|  | | | | | | | | | | **95% Credible Interval** | | | |  |
|  | | **N** | | **Mean** | | **SD** | | **SE** | | **Lower** | | **Upper** | |  |
| TRCE_mrt_effect |  | 75 |  | 297.099 |  | 243.610 |  | 28.130 |  | 241.049 |  | 353.149 |  |  |
| TRCE_effect_merr |  | 75 |  | 0.194 |  | 0.185 |  | 0.021 |  | 0.151 |  | 0.236 |  |  |
| BI_mrt_effect |  | 75 |  | 22.430 |  | 48.104 |  | 5.555 |  | 11.363 |  | 33.498 |  |  |
| BI_effect_merr |  | 75 |  | 0.003 |  | 0.023 |  | 0.003 |  | -0.003 |  | 0.008 |  |  |
| crs_mrt_effect |  | 75 |  | 37.931 |  | 81.023 |  | 9.356 |  | 19.290 |  | 56.573 |  |  |
| crs_effect_merr |  | 75 |  | 0.012 |  | 0.032 |  | 0.004 |  | 0.005 |  | 0.019 |  |  |
|  | | | | | | | | | | | | | |  |

Outcome neutral criteria were met in all criteria except for the BI in errors. Because we were mostly interested in RT, we ran the analyses.

Both hypotheses were directional but in opposite directions:

**For the TRCE**, we predicted that the TRCE will be reduced in the pro- compared to the anti- free will condition. This pattern was expected both in the control-prediction and in the automaticity prediction:

**Bayesian Independent Samples T-Test:TRCE**

| **Bayesian Independent Samples T-Test** | | | | | | | | | | | | | | | | |
| --- | --- | --- | --- | --- | --- | --- | --- | --- | --- | --- | --- | --- | --- | --- | --- | --- |
|  | | | | | | | | | | | **BF₀₊** | | | | **error %** | |
| TRCE_mrt_effect | | | | | | | | | |  | 2.854 | | |  | ~ 2.877e-5 |  |
| TRCE_effect_merr | | | | | | | | | |  | 20.432 | | |  | ~ 0.002 |  |
|  | | | | | | | | | | | | | | | | |
| *Note.*  For all tests, the alternative hypothesis specifies that the location of group *anti* is greater than the location of group *pro* . | | | | | | | | | | | | | | | | |
| **Descriptives** | | | | | | | | | | | | | | | |  |
|  | | | | | | | | | | | | **95% Credible Interval** | | | |  |
|  | | **Group** | | **N** | | **Mean** | | **SD** | | **SE** | | **Lower** | | **Upper** | |  |
| TRCE_mrt_effect |  | anti |  | 84 |  | 340.709 |  | 281.710 |  | 30.737 |  | 279.574 |  | 401.844 |  |  |
|  |  | pro |  | 75 |  | 297.099 |  | 243.610 |  | 28.130 |  | 241.049 |  | 353.149 |  |  |
| TRCE_effect_merr |  | anti |  | 84 |  | 0.150 |  | 0.148 |  | 0.016 |  | 0.118 |  | 0.182 |  |  |
|  |  | pro |  | 75 |  | 0.194 |  | 0.185 |  | 0.021 |  | 0.151 |  | 0.236 |  |  |
|  | | | | | | | | | | | | | | | |  |

**For the Bi & CRS effects**, we had two predictions. According to the control prediction, we predicted that the effects will be enhanced in the pro- compared to the anti- free will condition. According to the automaticity prediction, we predicted that it will not differ. We thus tested the directional hypothesis for both predictions:

**Bayesian Independent Samples T-Test: BI & CRS**

| **Bayesian Independent Samples T-Test** | | | | | | | | | | | | | | | | |  | |  | |  | |  | |
| --- | --- | --- | --- | --- | --- | --- | --- | --- | --- | --- | --- | --- | --- | --- | --- | --- | --- | --- | --- | --- | --- | --- | --- | --- |
|  | | | | | | | | | | | **BF₀₋** | | | | **error %** | |  | |  | |  | |  | |
| BI_mrt_effect | | | | | | | | | |  | 14.689 | | |  | ~ 0.029 |  |  | |  | |  | |  | |
| BI_effect_merr | | | | | | | | | |  | 1.297 | | |  | ~ 1.212e-4 |  |  | |  | |  | |  | |
| crs_mrt_effect | | | | | | | | | |  | 2.782 | | |  | ~ 2.750e-5 |  |  | |  | |  | |  | |
| crs_effect_merr | | | | | | | | | |  | 0.980 | | |  | ~ 1.857e-4 |  |  | |  | |  | |  | |
|  | | | | | | | | | | | | | | | | |  | |  | |  | |  | |
| *Note.*  For all tests, the alternative hypothesis specifies that the location of group *anti* is smaller than the location of group *pro* . | | | | | | | | | | | | | | | | |  | |  | |  | |  | |
| **Descriptives** | | | | | | | | | | | | | | | |  | |  | |  | |  | |  |
|  | | | | | | | | | | | | **95% Credible Interval** | | | |  | |  | |  | |  | |  |
|  | | **Group** | | **N** | | **Mean** | | **SD** | | **SE** | | **Lower** | | **Upper** | |  | |  | |  | |  | |  |
| BI_mrt_effect |  | anti |  | 84 |  | 29.934 |  | 53.051 |  | 5.788 |  | 18.421 |  | 41.447 |  |  | |  | |  | |  | |  |
|  |  | pro |  | 75 |  | 22.430 |  | 48.104 |  | 5.555 |  | 11.363 |  | 33.498 |  |  | |  | |  | |  | |  |
| BI_effect_merr |  | anti |  | 84 |  | -0.003 |  | 0.020 |  | 0.002 |  | -0.007 |  | 0.002 |  |  | |  | |  | |  | |  |
|  |  | pro |  | 75 |  | 0.003 |  | 0.023 |  | 0.003 |  | -0.003 |  | 0.008 |  |  | |  | |  | |  | |  |
| crs_mrt_effect |  | anti |  | 84 |  | 24.065 |  | 83.794 |  | 9.143 |  | 5.880 |  | 42.249 |  |  | |  | |  | |  | |  |
|  |  | pro |  | 75 |  | 37.931 |  | 81.023 |  | 9.356 |  | 19.290 |  | 56.573 |  |  | |  | |  | |  | |  |
| crs_effect_merr |  | anti |  | 84 |  | 0.004 |  | 0.026 |  | 0.003 |  | -0.002 |  | 0.010 |  |  | |  | |  | |  | |  |
|  |  | pro |  | 75 |  | 0.012 |  | 0.032 |  | 0.004 |  | 0.005 |  | 0.019 |  |  | |  | |  | |  | |  |
|  | | | | | | | | | | | | | | | |  | |  | |  | |  | |  |

We did not reach boundaries, and therefore we continued with data collection.

**Analysis 3**

**Outcome neutral – based on the pro free will group**

## Bayesian One Sample T-Test

| **Bayesian One Sample T-Test** | | | | | | | | | | | | | | |
| --- | --- | --- | --- | --- | --- | --- | --- | --- | --- | --- | --- | --- | --- | --- |
|  | | | | | | | | | **BF₊₀** | | | | **error %** | |
| TRCE_mrt_effect | | | | | | | |  | 8.133e+14 | | |  | NaN | ᵃ |
| TRCE_effect_merr | | | | | | | |  | 7.631e+14 | | |  | NaN | ᵃ |
| BI_mrt_effect | | | | | | | |  | 53.422 | | |  | ~ 1.967e-5 |  |
| BI_effect_merr | | | | | | | |  | 0.139 | | |  | ~ 5.075e-5 |  |
| crs_mrt_effect | | | | | | | |  | 180.562 | | |  | ~ 5.086e-8 |  |
| crs_effect_merr | | | | | | | |  | 72.630 | | |  | ~ 1.061e-4 |  |
|  | | | | | | | | | | | | | | |
| Note.  For all tests, the alternative hypothesis specifies that the population mean is greater than 0. | | | | | | | | | | | | | | |
| ᵃ t-value is large. A Savage-Dickey approximation was used to compute the Bayes factor but no error estimate can be given. | | | | | | | | | | | | | | |
| **Descriptives** | | | | | | | | | | | | | |  |
|  | | | | | | | | | | **95% Credible Interval** | | | |  |
|  | | **N** | | **Mean** | | **SD** | | **SE** | | **Lower** | | **Upper** | |  |
| TRCE_mrt_effect |  | 90 |  | 330.897 |  | 295.079 |  | 31.104 |  | 269.094 |  | 392.700 |  |  |
| TRCE_effect_merr |  | 90 |  | 0.197 |  | 0.176 |  | 0.019 |  | 0.160 |  | 0.233 |  |  |
| BI_mrt_effect |  | 90 |  | 21.022 |  | 56.496 |  | 5.955 |  | 9.189 |  | 32.855 |  |  |
| BI_effect_merr |  | 90 |  | 0.001 |  | 0.023 |  | 0.002 |  | -0.003 |  | 0.006 |  |  |
| crs_mrt_effect |  | 90 |  | 35.929 |  | 87.125 |  | 9.184 |  | 17.681 |  | 54.177 |  |  |
| crs_effect_merr |  | 90 |  | 0.012 |  | 0.031 |  | 0.003 |  | 0.005 |  | 0.018 |  |  |
|  | | | | | | | | | | | | | |  |

Outcome neutral criteria were met in all criteria except for the BI in errors. Because we were mostly interested in RT, we ran the analyses.

Both hypotheses were directional but in opposite directions:

**For the TRCE**, we predicted that the TRCE will be reduced in the pro- compared to the anti- free will condition. This pattern was expected both in the control-prediction and in the automaticity prediction:

**Bayesian Independent Samples T-Test:TRCE**

| **Bayesian Independent Samples T-Test** | | | | | | | | | | | | | | | | |
| --- | --- | --- | --- | --- | --- | --- | --- | --- | --- | --- | --- | --- | --- | --- | --- | --- |
|  | | | | | | | | | | | **BF₀₊** | | | | **error %** | |
| TRCE_mrt_effect | | | | | | | | | |  | 8.960 | | |  | ~ 9.517e-6 |  |
| TRCE_effect_merr | | | | | | | | | |  | 25.622 | | |  | ~ 0.006 |  |
|  | | | | | | | | | | | | | | | | |
| *Note.*  For all tests, the alternative hypothesis specifies that the location of group *anti* is greater than the location of group *pro* . | | | | | | | | | | | | | | | | |
| **Descriptives** | | | | | | | | | | | | | | | |  |
|  | | | | | | | | | | | | **95% Credible Interval** | | | |  |
|  | | **Group** | | **N** | | **Mean** | | **SD** | | **SE** | | **Lower** | | **Upper** | |  |
| TRCE_mrt_effect |  | anti |  | 99 |  | 329.826 |  | 271.899 |  | 27.327 |  | 275.596 |  | 384.055 |  |  |
|  |  | pro |  | 90 |  | 330.897 |  | 295.079 |  | 31.104 |  | 269.094 |  | 392.700 |  |  |
| TRCE_effect_merr |  | anti |  | 99 |  | 0.149 |  | 0.146 |  | 0.015 |  | 0.120 |  | 0.179 |  |  |
|  |  | pro |  | 90 |  | 0.197 |  | 0.176 |  | 0.019 |  | 0.160 |  | 0.233 |  |  |
|  | | | | | | | | | | | | | | | |  |

**For the Bi & CRS effects**, we had two predictions. According to the control prediction, we predicted that the effects will be enhanced in the pro- compared to the anti- free will condition. According to the automaticity prediction, we predicted that it will not differ. We thus tested the directional hypothesis for both predictions:

**Bayesian Independent Samples T-Test: BI & CRS**

| **Bayesian Independent Samples T-Test** | | | | | | | | | | | | | | | | |
| --- | --- | --- | --- | --- | --- | --- | --- | --- | --- | --- | --- | --- | --- | --- | --- | --- |
|  | | | | | | | | | | | **BF₀₋** | | | | **error %** | |
| BI_mrt_effect | | | | | | | | | |  | 14.915 | | |  | ~ 0.116 |  |
| BI_effect_merr | | | | | | | | | |  | 2.283 | | |  | ~ 9.615e-5 |  |
| crs_mrt_effect | | | | | | | | | |  | 2.722 | | |  | ~ 8.233e-5 |  |
| crs_effect_merr | | | | | | | | | |  | 1.219 | | |  | ~ 9.052e-6 |  |
|  | | | | | | | | | | | | | | | | |
| *Note.*  For all tests, the alternative hypothesis specifies that the location of group *anti* is smaller than the location of group *pro* . | | | | | | | | | | | | | | | | |
| **Descriptives** | | | | | | | | | | | | | | | |  |
|  | | | | | | | | | | | | **95% Credible Interval** | | | |  |
|  | | **Group** | | **N** | | **Mean** | | **SD** | | **SE** | | **Lower** | | **Upper** | |  |
| BI_mrt_effect |  | anti |  | 99 |  | 27.312 |  | 51.426 |  | 5.168 |  | 17.055 |  | 37.569 |  |  |
|  |  | pro |  | 90 |  | 21.022 |  | 56.496 |  | 5.955 |  | 9.189 |  | 32.855 |  |  |
| BI_effect_merr |  | anti |  | 99 |  | -0.003 |  | 0.020 |  | 0.002 |  | -0.007 |  | 0.001 |  |  |
|  |  | pro |  | 90 |  | 0.001 |  | 0.023 |  | 0.002 |  | -0.003 |  | 0.006 |  |  |
| crs_mrt_effect |  | anti |  | 99 |  | 21.625 |  | 85.988 |  | 8.642 |  | 4.475 |  | 38.775 |  |  |
|  |  | pro |  | 90 |  | 35.929 |  | 87.125 |  | 9.184 |  | 17.681 |  | 54.177 |  |  |
| crs_effect_merr |  | anti |  | 99 |  | 0.005 |  | 0.028 |  | 0.003 |  | -9.738e-4 |  | 0.010 |  |  |
|  |  | pro |  | 90 |  | 0.012 |  | 0.031 |  | 0.003 |  | 0.005 |  | 0.018 |  |  |
|  | | | | | | | | | | | | | | | |  |

We did not reach boundaries, and therefore we continued with data collection.

**Analysis 4**

**Outcome neutral – based on the entire sample**

**Bayesian One Sample T-Test**

| **Bayesian One Sample T-Test** | | | | | | | | | | | | | | |
| --- | --- | --- | --- | --- | --- | --- | --- | --- | --- | --- | --- | --- | --- | --- |
|  | | | | | | | | | **BF₊₀** | | | | **error %** | |
| TRCE_mrt_effect | | | | | | | |  | 1.306e+38 | | |  | NaN | ᵃ |
| TRCE_effect_merr | | | | | | | |  | 1.004e+32 | | |  | NaN | ᵃ |
| BI_mrt_effect | | | | | | | |  | 1.905e+8 | | |  | NaN | ᵃ |
| BI_effect_merr | | | | | | | |  | 0.045 | | |  | ~ 0.002 |  |
| crs_mrt_effect | | | | | | | |  | 2889.560 | | |  | ~ 8.374e-8 |  |
| crs_effect_merr | | | | | | | |  | 105.496 | | |  | ~ 1.145e-5 |  |
|  | | | | | | | | | | | | | | |
| *Note.*  For all tests, the alternative hypothesis specifies that the population mean is greater than 0. | | | | | | | | | | | | | | |
| ᵃ t-value is large. A Savage-Dickey approximation was used to compute the Bayes factor but no error estimate can be given. | | | | | | | | | | | | | | |
| **Descriptives** | | | | | | | | | | | | | |  |
|  | | | | | | | | | | **95% Credible Interval** | | | |  |
|  | | **N** | | **Mean** | | **SD** | | **SE** | | **Lower** | | **Upper** | |  |
| TRCE_mrt_effect |  | 204 |  | 338.048 |  | 282.733 |  | 19.795 |  | 299.017 |  | 377.078 |  |  |
| TRCE_effect_merr |  | 204 |  | 0.174 |  | 0.165 |  | 0.012 |  | 0.151 |  | 0.197 |  |  |
| BI_mrt_effect |  | 204 |  | 25.641 |  | 53.107 |  | 3.718 |  | 18.310 |  | 32.972 |  |  |
| BI_effect_merr |  | 204 |  | -4.187e-4 |  | 0.021 |  | 0.001 |  | -0.003 |  | 0.002 |  |  |
| crs_mrt_effect |  | 204 |  | 27.905 |  | 85.977 |  | 6.020 |  | 16.036 |  | 39.774 |  |  |
| crs_effect_merr |  | 204 |  | 0.008 |  | 0.029 |  | 0.002 |  | 0.004 |  | 0.012 |  |  |
|  | | | | | | | | | | | | | |  |

**Outcome neutral – based on the pro free will group**

## Bayesian One Sample T-Test

| **Bayesian One Sample T-Test** | | | | | | | | | | | | | | |
| --- | --- | --- | --- | --- | --- | --- | --- | --- | --- | --- | --- | --- | --- | --- |
|  | | | | | | | | | **BF₊₀** | | | | **error %** | |
| TRCE_mrt_effect | | | | | | | |  | 1.571e+18 | | |  | NaN | ᵃ |
| TRCE_effect_merr | | | | | | | |  | 3.778e+16 | | |  | NaN | ᵃ |
| BI_mrt_effect | | | | | | | |  | 1118.900 | | |  | ~ 9.035e-9 |  |
| BI_effect_merr | | | | | | | |  | 0.150 | | |  | ~ 5.050e-5 |  |
| crs_mrt_effect | | | | | | | |  | 278.601 | | |  | ~ 6.296e-8 |  |
| crs_effect_merr | | | | | | | |  | 53.411 | | |  | ~ 5.483e-8 |  |
|  | | | | | | | | | | | | | | |
| Note.  For all tests, the alternative hypothesis specifies that the population mean is greater than 0. | | | | | | | | | | | | | | |
| ᵃ t-value is large. A Savage-Dickey approximation was used to compute the Bayes factor but no error estimate can be given. | | | | | | | | | | | | | | |
| **Descriptives** | | | | | | | | | | | | | |  |
|  | | | | | | | | | | **95% Credible Interval** | | | |  |
|  | | **N** | | **Mean** | | **SD** | | **SE** | | **Lower** | | **Upper** | |  |
| TRCE_mrt_effect |  | 101 |  | 342.770 |  | 289.182 |  | 28.775 |  | 285.682 |  | 399.858 |  |  |
| TRCE_effect_merr |  | 101 |  | 0.193 |  | 0.174 |  | 0.017 |  | 0.159 |  | 0.227 |  |  |
| BI_mrt_effect |  | 101 |  | 24.467 |  | 55.529 |  | 5.525 |  | 13.504 |  | 35.429 |  |  |
| BI_effect_merr |  | 101 |  | 0.002 |  | 0.022 |  | 0.002 |  | -0.003 |  | 0.006 |  |  |
| crs_mrt_effect |  | 101 |  | 35.005 |  | 87.144 |  | 8.671 |  | 17.802 |  | 52.209 |  |  |
| crs_effect_merr |  | 101 |  | 0.011 |  | 0.031 |  | 0.003 |  | 0.005 |  | 0.017 |  |  |
|  | | | | | | | | | | | | | |  |

**Main analyses**

Outcome neutral criteria were met in all criteria except for the BI in errors. Because we were mostly interested in RT, we ran the analyses.

Both hypotheses were directional but in opposite directions:

**For the TRCE**, we predicted that the TRCE will be reduced in the pro- compared to the anti- free will condition. This pattern was expected both in the control-prediction and in the automaticity prediction:

**Bayesian Independent Samples T-Test: TRCE**

| **Bayesian Independent Samples T-Test** | | | | | | | | | | | | | | | | |
| --- | --- | --- | --- | --- | --- | --- | --- | --- | --- | --- | --- | --- | --- | --- | --- | --- |
|  | | | | | | | | | | | **BF₀₊** | | | | **error %** | |
| TRCE_mrt_effect | | | | | | | | | |  | 10.868 | | |  | ~ 0.037 |  |
| TRCE_effect_merr | | | | | | | | | |  | 23.343 | | |  | ~ 0.017 |  |
|  | | | | | | | | | | | | | | | | |
| *Note.*  For all tests, the alternative hypothesis specifies that the location of group *anti* is greater than the location of group *pro* . | | | | | | | | | | | | | | | | |
| **Descriptives** | | | | | | | | | | | | | | | |  |
|  | | | | | | | | | | | | **95% Credible Interval** | | | |  |
|  | | **Group** | | **N** | | **Mean** | | **SD** | | **SE** | | **Lower** | | **Upper** | |  |
| TRCE_mrt_effect |  | anti |  | 103 |  | 333.417 |  | 277.601 |  | 27.353 |  | 279.163 |  | 387.671 |  |  |
|  |  | pro |  | 101 |  | 342.770 |  | 289.182 |  | 28.775 |  | 285.682 |  | 399.858 |  |  |
| TRCE_effect_merr |  | anti |  | 103 |  | 0.155 |  | 0.153 |  | 0.015 |  | 0.125 |  | 0.185 |  |  |
|  |  | pro |  | 101 |  | 0.193 |  | 0.174 |  | 0.017 |  | 0.159 |  | 0.227 |  |  |
|  | | | | | | | | | | | | | | | |  |

**For the Bi & CRS effects**, we had two predictions. According to the control prediction, we predicted that the effects will be enhanced in the pro- compared to the anti- free will condition. According to the automaticity prediction, we predicted that it will not differ. We thus tested the directional hypothesis for both predictions:

**Bayesian Independent Samples T-Test: BI & CRS**

| **Bayesian Independent Samples T-Test** | | | | | | | | | | | | | | | | |
| --- | --- | --- | --- | --- | --- | --- | --- | --- | --- | --- | --- | --- | --- | --- | --- | --- |
|  | | | | | | | | | | | **BF₀₋** | | | | **error %** | |
| BI_mrt_effect | | | | | | | | | |  | 11.461 | | |  | ~ 0.103 |  |
| BI_effect_merr | | | | | | | | | |  | 2.247 | | |  | ~ 1.181e-4 |  |
| crs_mrt_effect | | | | | | | | | |  | 2.695 | | |  | ~ 6.776e-5 |  |
| crs_effect_merr | | | | | | | | | |  | 1.713 | | |  | ~ 1.499e-4 |  |
|  | | | | | | | | | | | | | | | | |
| *Note.*  For all tests, the alternative hypothesis specifies that the location of group *anti* is smaller than the location of group *pro* . | | | | | | | | | | | | | | | | |
| **Descriptives** | | | | | | | | | | | | | | | |  |
|  | | | | | | | | | | | | **95% Credible Interval** | | | |  |
|  | | **Group** | | **N** | | **Mean** | | **SD** | | **SE** | | **Lower** | | **Upper** | |  |
| BI_mrt_effect |  | anti |  | 103 |  | 26.793 |  | 50.866 |  | 5.012 |  | 16.852 |  | 36.734 |  |  |
|  |  | pro |  | 101 |  | 24.467 |  | 55.529 |  | 5.525 |  | 13.504 |  | 35.429 |  |  |
| BI_effect_merr |  | anti |  | 103 |  | -0.002 |  | 0.020 |  | 0.002 |  | -0.006 |  | 0.002 |  |  |
|  |  | pro |  | 101 |  | 0.002 |  | 0.022 |  | 0.002 |  | -0.003 |  | 0.006 |  |  |
| crs_mrt_effect |  | anti |  | 103 |  | 20.943 |  | 84.662 |  | 8.342 |  | 4.397 |  | 37.490 |  |  |
|  |  | pro |  | 101 |  | 35.005 |  | 87.144 |  | 8.671 |  | 17.802 |  | 52.209 |  |  |
| crs_effect_merr |  | anti |  | 103 |  | 0.005 |  | 0.028 |  | 0.003 |  | -6.560e-4 |  | 0.010 |  |  |
|  |  | pro |  | 101 |  | 0.011 |  | 0.031 |  | 0.003 |  | 0.005 |  | 0.017 |  |  |
|  | | | | | | | | | | | | | | | |  |

We did not reach boundaries, but we reached n.max.

**Correlations**

With FWI – only pro group:

Expecting a negative correlation with TRCE (test for a negative correlation):

| **Bayesian Pearson Correlations** | | | | | | | | | |
| --- | --- | --- | --- | --- | --- | --- | --- | --- | --- |
| **Variable** | |  | | **FWI** | | **------------------------** | | **------------** | |
| 1. FWI |  | n |  | — |  |  |  |  |  |
|  |  | Pearson's r |  | — |  |  |  |  |  |
|  |  | BF₀₁ |  | — |  |  |  |  |  |
| 2. TRCE_mrt_effect |  | n |  | 101.000 |  |  |  |  |  |
|  |  | Pearson's r |  | 0.017 |  |  |  |  |  |
|  |  | BF₀₁ |  | 9.133 |  |  |  |  |  |
| 3. TRCE_effect_merr |  | n |  | 101.000 |  |  |  |  |  |
|  |  | Pearson's r |  | 0.033 |  |  |  |  |  |
|  |  | BF₀₁ |  | 10.214 |  |  |  |  |  |
|  | | | | | | | | | |
| *Note.*  For all tests, the alternative hypothesis specifies that the correlation is negative | | | | | | | | | |

Expecting positive correlation with BI and CRS:

**BI & CRS: Bayesian Correlation**

| **Bayesian Pearson Correlations** | | | | | | | | | | | | | |
| --- | --- | --- | --- | --- | --- | --- | --- | --- | --- | --- | --- | --- | --- |
| **Variable** | |  | | **FWI** | | **-------------** | | **-------------** | | **-------------** | | **-------------** | |
| 1. FWI |  | n |  | — |  |  |  |  |  |  |  |  |  |
|  |  | Pearson's r |  | — |  |  |  |  |  |  |  |  |  |
|  |  | BF₀₊ |  | — |  |  |  |  |  |  |  |  |  |
| 2. BI_mrt_effect |  | n |  | 101.000 |  |  |  |  |  |  |  |  |  |
|  |  | Pearson's r |  | 0.043 |  |  |  |  |  |  |  |  |  |
|  |  | BF₀₊ |  | 5.523 |  |  |  |  |  |  |  |  |  |
| 3. BI_effect_merr |  | n |  | 101.000 |  |  |  |  |  |  |  |  |  |
|  |  | Pearson's r |  | -0.116 |  |  |  |  |  |  |  |  |  |
|  |  | BF₀₊ |  | 16.479 |  |  |  |  |  |  |  |  |  |
| 4. crs_mrt_effect |  | n |  | 101.000 |  |  |  |  |  |  |  |  |  |
|  |  | Pearson's r |  | -0.063 |  |  |  |  |  |  |  |  |  |
|  |  | BF₀₊ |  | 12.391 |  |  |  |  |  |  |  |  |  |
| 5. crs_effect_merr |  | n |  | 101.000 |  |  |  |  |  |  |  |  |  |
|  |  | Pearson's r |  | 0.073 |  |  |  |  |  |  |  |  |  |
|  |  | BF₀₊ |  | 4.079 |  |  |  |  |  |  |  |  |  |
|  | | | | | | | | | | | | | |
| *Note.*  For all tests, the alternative hypothesis specifies that the correlation is positive. | | | | | | | | | | | | | |

Entire sample, FWI:

Expecting a negative correlation with TRCE (test for a negative correlation):

**FWI - Bayesian Correlation: TRCE**

| **Bayesian Pearson Correlations** | | | | | | | | | |
| --- | --- | --- | --- | --- | --- | --- | --- | --- | --- |
| **Variable** | |  | | **FWI** | | **-------------------------** | | **---------------------** | |
| 1. FWI |  | n |  | — |  |  |  |  |  |
|  |  | Pearson's r |  | — |  |  |  |  |  |
|  |  | BF₀₋ |  | — |  |  |  |  |  |
| 2. TRCE_mrt_effect |  | n |  | 204.000 |  |  |  |  |  |
|  |  | Pearson's r |  | -0.001 |  |  |  |  |  |
|  |  | BF₀₋ |  | 11.239 |  |  |  |  |  |
| 3. TRCE_effect_merr |  | n |  | 204.000 |  |  |  |  |  |
|  |  | Pearson's r |  | 0.097 |  |  |  |  |  |
|  |  | BF₀₋ |  | 26.195 |  |  |  |  |  |
|  | | | | | | | | | |
| *Note.*  For all tests, the alternative hypothesis specifies that the correlation is negative | | | | | | | | | |

Expecting positive correlation with BI and CRS:

**FWI - Bayesian Correlation: BI & CRS**

| **Bayesian Pearson Correlations** | | | | | | | | | | | | | |
| --- | --- | --- | --- | --- | --- | --- | --- | --- | --- | --- | --- | --- | --- |
| **Variable** | |  | | **FWI** | | **------------** | | **-----------** | | **-----------** | | **-----------** | |
| 1. FWI |  | n |  | — |  |  |  |  |  |  |  |  |  |
|  |  | Pearson's r |  | — |  |  |  |  |  |  |  |  |  |
|  |  | BF₀₊ |  | — |  |  |  |  |  |  |  |  |  |
| 2. BI_mrt_effect |  | n |  | 204.000 |  |  |  |  |  |  |  |  |  |
|  |  | Pearson's r |  | 0.005 |  |  |  |  |  |  |  |  |  |
|  |  | BF₀₊ |  | 10.734 |  |  |  |  |  |  |  |  |  |
| 3. BI_effect_merr |  | n |  | 204.000 |  |  |  |  |  |  |  |  |  |
|  |  | Pearson's r |  | 0.007 |  |  |  |  |  |  |  |  |  |
|  |  | BF₀₊ |  | 10.578 |  |  |  |  |  |  |  |  |  |
| 4. crs_mrt_effect |  | n |  | 204.000 |  |  |  |  |  |  |  |  |  |
|  |  | Pearson's r |  | 0.035 |  |  |  |  |  |  |  |  |  |
|  |  | BF₀₊ |  | 7.339 |  |  |  |  |  |  |  |  |  |
| 5. crs_effect_merr |  | n |  | 204.000 |  |  |  |  |  |  |  |  |  |
|  |  | Pearson's r |  | 0.175 |  |  |  |  |  |  |  |  |  |
|  |  | BF₀₊ |  | 0.252 |  |  |  |  |  |  |  |  |  |
|  | | | | | | | | | | | | | |
| *Note.*  For all tests, the alternative hypothesis specifies that the correlation is positive. | | | | | | | | | | | | | |

### Because the correlation with CRS-PE is substantial, we ran further analyses:

### Bayesian Correlation Pairwise Plots

#### FWI - crs_effect_merr

##### Scatterplot


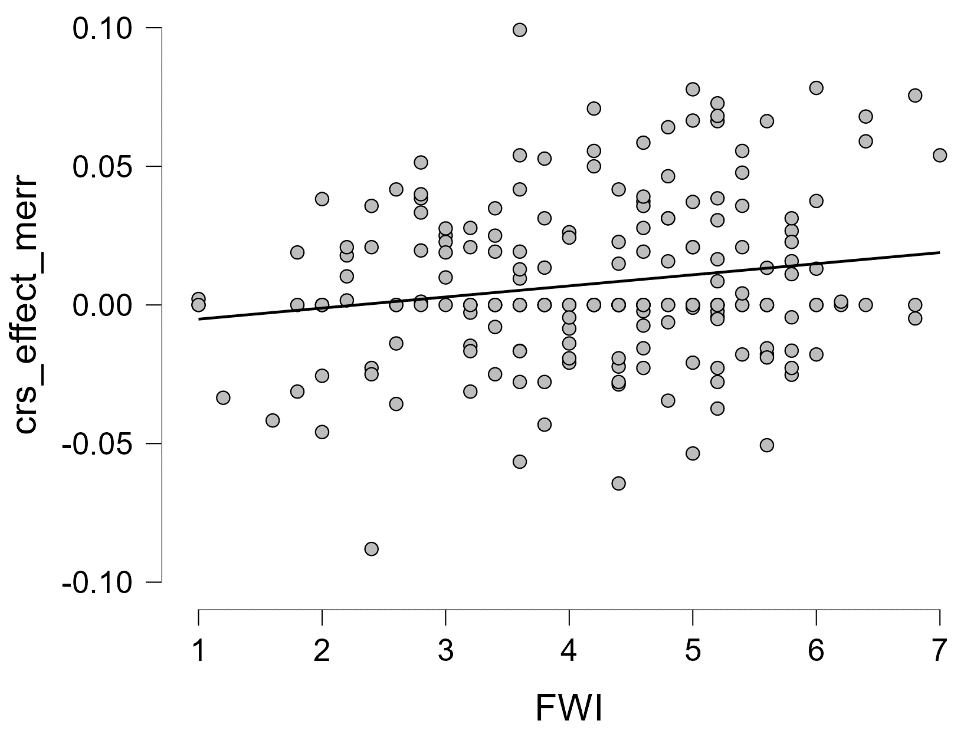


##### Bayes Factor Robustness Check


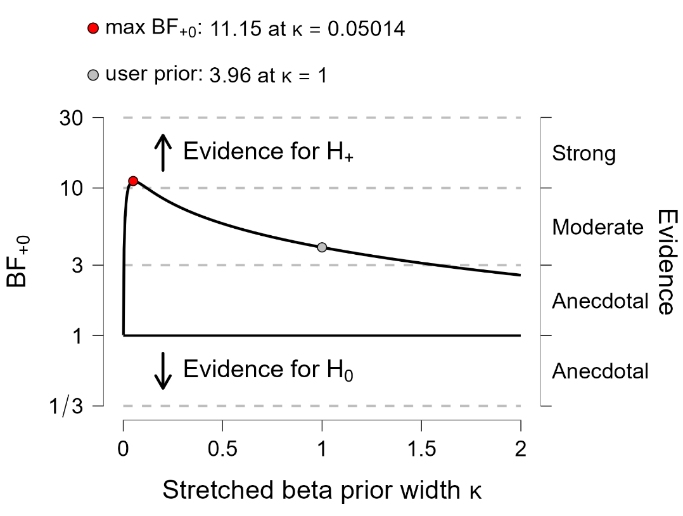


##### Sequential Analysis


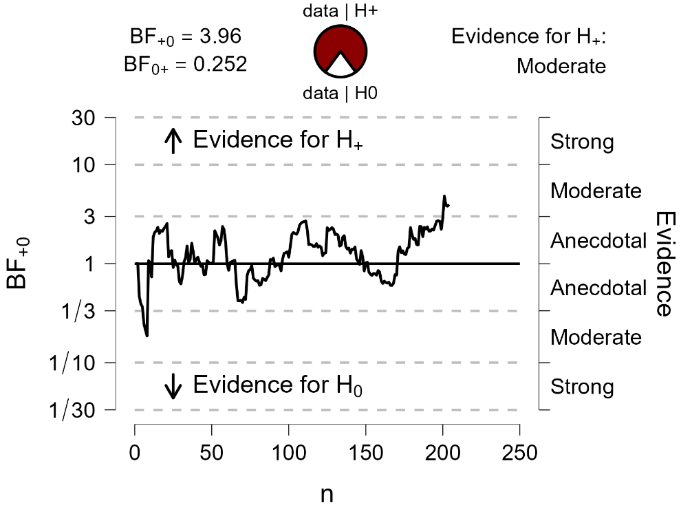


The robustness check (i.e., the extent to which the results depend on the priors) of this correlation in the sample with exclusions indicate that the effect would have been substantial (but hardly ever strong) in a large range of priors, yet a sequential analysis indicates that the effect might be anecdotal.

Entire sample, LOC, no clear predictions:

**LoC: Bayesian Correlation - Exploratory**

| **Variable** | |  | | **LoC** | |
| --- | --- | --- | --- | --- | --- |
| 1. LoC |  | Pearson's r |  | — |  |
|  |  | BF₁₀ |  | — |  |
| 2. FWI |  | Pearson's r |  | 0.384 |  |
|  |  | BF₁₀ |  | 714168.076 |  |
| 3. TRCE_mrt_effect |  | Pearson's r |  | 0.021 |  |
|  |  | BF₁₀ |  | 0.092 |  |
| 4. TRCE_effect_merr |  | Pearson's r |  | -0.038 |  |
|  |  | BF₁₀ |  | 0.101 |  |
| 5. BI_mrt_effect |  | Pearson's r |  | 0.055 |  |
|  |  | BF₁₀ |  | 0.119 |  |
| 6. BI_effect_merr |  | Pearson's r |  | -0.145 |  |
|  |  | BF₁₀ |  | 0.736 |  |
| 7. crs_mrt_effect |  | Pearson's r |  | 0.105 |  |
|  |  | BF₁₀ |  | 0.265 |  |
| 8. crs_effect_merr |  | Pearson's r |  | 0.032 |  |
|  |  | BF₁₀ |  | 0.097 |  |

**Pre-registered regressions**

## TRCE-RT Bayesian Linear Regression - LoC

| **Model Comparison - TRCE_mrt_effect** | | | | | | | | | | | |
| --- | --- | --- | --- | --- | --- | --- | --- | --- | --- | --- | --- |
| **Models** | | **P(M)** | | **P(M\|data)** | | **BF_M_** | | **BF_10_** | | **R²** | |
| Null model |  | 0.333 |  | 0.837 |  | 10.259 |  | 1.000 |  | 0.000 |  |
| LoC |  | 0.167 |  | 0.066 |  | 0.356 |  | 0.159 |  | 0.000 |  |
| cond_pro0.5antiminus0.5 |  | 0.167 |  | 0.065 |  | 0.350 |  | 0.156 |  | 0.000 |  |
| cond_pro0.5antiminus0.5 + LoC |  | 0.333 |  | 0.031 |  | 0.065 |  | 0.037 |  | 0.001 |  |
|  | | | | | | | | | | | |

## TRCE-PE Bayesian Linear Regression - LoC

| **Model Comparison - TRCE_effect_merr** | | | | | | | | | | | |
| --- | --- | --- | --- | --- | --- | --- | --- | --- | --- | --- | --- |
| **Models** | | **P(M)** | | **P(M\|data)** | | **BF_M_** | | **BF_10_** | | **R²** | |
| Null model |  | 0.333 |  | 0.660 |  | 3.887 |  | 1.000 |  | 0.000 |  |
| cond_pro0.5antiminus0.5 |  | 0.167 |  | 0.183 |  | 1.120 |  | 0.554 |  | 0.014 |  |
| cond_pro0.5antiminus0.5 + LoC |  | 0.333 |  | 0.099 |  | 0.220 |  | 0.150 |  | 0.016 |  |
| LoC |  | 0.167 |  | 0.058 |  | 0.305 |  | 0.174 |  | 0.001 |  |
|  | | | | | | | | | | | |

## BI-RT Bayesian Linear Regression - LoC

| **Model Comparison - BI_mrt_effect** | | | | | | | | | | | |
| --- | --- | --- | --- | --- | --- | --- | --- | --- | --- | --- | --- |
| **Models** | | **P(M)** | | **P(M\|data)** | | **BF_M_** | | **BF_10_** | | **R²** | |
| Null model |  | 0.333 |  | 0.813 |  | 8.678 |  | 1.000 |  | 0.000 |  |
| LoC |  | 0.167 |  | 0.082 |  | 0.449 |  | 0.203 |  | 0.003 |  |
| cond_pro0.5antiminus0.5 |  | 0.167 |  | 0.065 |  | 0.346 |  | 0.159 |  | 0.000 |  |
| cond_pro0.5antiminus0.5 + LoC |  | 0.333 |  | 0.040 |  | 0.084 |  | 0.049 |  | 0.004 |  |
|  | | | | | | | | | | | |

## CRS-RT Bayesian Linear Regression - LoC

| **Model Comparison - crs_mrt_effect** | | | | | | | | | | | |
| --- | --- | --- | --- | --- | --- | --- | --- | --- | --- | --- | --- |
| **Models** | | **P(M)** | | **P(M\|data)** | | **BF_M_** | | **BF_10_** | | **R²** | |
| Null model |  | 0.333 |  | 0.654 |  | 3.786 |  | 1.000 |  | 0.000 |  |
| LoC |  | 0.167 |  | 0.143 |  | 0.831 |  | 0.436 |  | 0.011 |  |
| cond_pro0.5antiminus0.5 + LoC |  | 0.333 |  | 0.109 |  | 0.244 |  | 0.166 |  | 0.017 |  |
| cond_pro0.5antiminus0.5 |  | 0.167 |  | 0.094 |  | 0.521 |  | 0.289 |  | 0.007 |  |
|  | | | | | | | | | | | |

## CRS-PE Bayesian Linear Regression - LoC

| **Model Comparison - crs_effect_merr** | | | | | | | | | | | |
| --- | --- | --- | --- | --- | --- | --- | --- | --- | --- | --- | --- |
| **Models** | | **P(M)** | | **P(M\|data)** | | **BF_M_** | | **BF_10_** | | **R²** | |
| Null model |  | 0.333 |  | 0.716 |  | 5.039 |  | 1.000 |  | 0.000 |  |
| cond_pro0.5antiminus0.5 |  | 0.167 |  | 0.152 |  | 0.893 |  | 0.424 |  | 0.011 |  |
| cond_pro0.5antiminus0.5 + LoC |  | 0.333 |  | 0.072 |  | 0.156 |  | 0.101 |  | 0.011 |  |
| LoC |  | 0.167 |  | 0.060 |  | 0.320 |  | 0.168 |  | 0.001 |  |
|  | | | | | | | | | | | |

**Further t-tests**

The impact the manipulation had on: FWI (free will inventory, LoC (locus of control), subjective fatigue, and RT (mrt).

**Impact of the manipulation on the FWI manipulation**

**Bayesian Independent Samples T-Test: FWI**

| **Bayesian Independent Samples T-Test** | | | | | | | | | | | | |  |  |  |
| --- | --- | --- | --- | --- | --- | --- | --- | --- | --- | --- | --- | --- | --- | --- | --- |
|  | | | **BF₁₀** | | | | | | **error %** | | | |  |  |  |
| FWI | |  | 11848.593 | | | | |  | 2.932e-10 | | |  |  |  |  |
|  | | | | | | | | | | | | |  |  |  |
| **Descriptives** | | | | | | | | | | | | | | | |
|  | | | | | | | | | | | | **95% Credible Interval** | | | |
|  | | **Group** | | **N** | | **Mean** | | **SD** | | **SE** | | **Lower** | | **Upper** | |
| FWI |  | anti |  | 103 |  | 3.792 |  | 1.225 |  | 0.121 |  | 3.553 |  | 4.032 |  |
|  |  | pro |  | 101 |  | 4.646 |  | 1.199 |  | 0.119 |  | 4.409 |  | 4.882 |  |
|  | | | | | | | | | | | | | | | |

**Impact on fatigue & LoC:**

**Bayesian Independent Samples T-Test: Fatigue & LoC**

| **Bayesian Independent Samples T-Test** | | | | | | | | |  |  |  |  |  |  |  |
| --- | --- | --- | --- | --- | --- | --- | --- | --- | --- | --- | --- | --- | --- | --- | --- |
|  | | | **BF₀₁** | | **error %** | | | |  |  |  |  |  |  |  |
| subjective_fatigue | |  | 3.723 |  | 3.639e-6 | | |  |  |  |  |  |  |  |  |
| mrt | |  | 5.405 |  | 4.612e-6 | | |  |  |  |  |  |  |  |  |
| LoC | |  | 5.898 |  | 4.864e-6 | | |  |  |  |  |  |  |  |  |
|  | | | | | | | | |  |  |  |  |  |  |  |
| **Descriptives** | | | | | | | | | | | | | | | |
|  | | | | | | | | | | | | **95% Credible Interval** | | | |
|  | | **Group** | | **N** | | **Mean** | | **SD** | | **SE** | | **Lower** | | **Upper** | |
| subjective_fatigue |  | anti |  | 103 |  | 3.522 |  | 1.094 |  | 0.108 |  | 3.308 |  | 3.736 |  |
|  |  | pro |  | 101 |  | 3.307 |  | 1.159 |  | 0.115 |  | 3.078 |  | 3.536 |  |
| mrt |  | anti |  | 103 |  | 922.782 |  | 262.091 |  | 25.825 |  | 871.559 |  | 974.005 |  |
|  |  | pro |  | 101 |  | 887.327 |  | 222.649 |  | 22.154 |  | 843.374 |  | 931.281 |  |
| LoC |  | anti |  | 103 |  | 8.563 |  | 4.383 |  | 0.432 |  | 7.707 |  | 9.420 |  |
|  |  | pro |  | 101 |  | 9.149 |  | 4.424 |  | 0.440 |  | 8.275 |  | 10.022 |  |
|  | | | | | | | | | | | | | | | |

**No Exclusions Analysis**

**Outcome neutral – based on the entire sample**

**Bayesian One Sample T-Test**

| **Bayesian One Sample T-Test** | | | | | | | | | | | | | | | | |
| --- | --- | --- | --- | --- | --- | --- | --- | --- | --- | --- | --- | --- | --- | --- | --- | --- |
|  | | | | | | | | | **BF₊₀** | | | | | | **error %** | |
| TRCE_mrt_effect | | | | | | | |  | 2.250e+83 | | | | |  | NaN | ᵃ |
| TRCE_effect_merr | | | | | | | |  | 3.525e+71 | | | | |  | NaN | ᵃ |
| BI_mrt_effect | | | | | | | |  | 33072.086 | | | | |  | NaN | ᵃ |
| BI_effect_merr | | | | | | | |  | 0.016 | | | | |  | ~ 0.672 |  |
| crs_mrt_effect | | | | | | | |  | 5.389e+17 | | | | |  | NaN | ᵃ |
| crs_effect_merr | | | | | | | |  | 215401.944 | | | | |  | NaN | ᵃ |
|  | | | | | | | | | | | | | | | | |
| *Note.*  For all tests, the alternative hypothesis specifies that the population mean is greater than 0. | | | | | | | | | | | | | | | | |
| ᵃ t-value is large. A Savage-Dickey approximation was used to compute the Bayes factor but no error estimate can be given. | | | | | | | | | | | | | | | | |
| **Descriptives** | | | | | | | | | | | | | |  |  |  |
|  | | | | | | | | | | **95% Credible Interval** | | | |  |  |  |
|  | | **N** | | **Mean** | | **SD** | | **SE** | | **Lower** | | **Upper** | |  |  |  |
| TRCE_mrt_effect |  | 567 |  | 368.000 |  | 369.880 |  | 15.533 |  | 337.490 |  | 398.511 |  |  |  |  |
| TRCE_effect_merr |  | 581 |  | 0.305 |  | 0.345 |  | 0.014 |  | 0.277 |  | 0.333 |  |  |  |  |
| BI_mrt_effect |  | 586 |  | 13.235 |  | 61.774 |  | 2.552 |  | 8.223 |  | 18.247 |  |  |  |  |
| BI_effect_merr |  | 586 |  | -0.001 |  | 0.030 |  | 0.001 |  | -0.004 |  | 9.903e-4 |  |  |  |  |
| crs_mrt_effect |  | 571 |  | 39.963 |  | 98.362 |  | 4.116 |  | 31.878 |  | 48.048 |  |  |  |  |
| crs_effect_merr |  | 574 |  | 0.012 |  | 0.054 |  | 0.002 |  | 0.008 |  | 0.017 |  |  |  |  |
|  | | | | | | | | | | | | | |  |  |  |

**Outcome neutral – based on the pro free will group**

| **Bayesian One Sample T-Test** | | | | | |
| --- | --- | --- | --- | --- | --- |
|  | | **BF₊₀** | | **error %** | |
| TRCE_mrt_effect |  | 1.163e+44 |  | NaN | ᵃ |
| TRCE_effect_merr |  | 5.686e+38 |  | NaN | ᵃ |
| BI_mrt_effect |  | 77.483 |  | ~ 9.861e-5 |  |
| BI_effect_merr |  | 0.019 |  | ~ 8.160e-4 |  |
| crs_mrt_effect |  | 3.644e+10 |  | NaN | ᵃ |
| crs_effect_merr |  | 1029.746 |  | ~ 4.841e-7 |  |
|  | | | | | |
| *Note.*  For all tests, the alternative hypothesis specifies that the population mean is greater than 0. | | | | | |
| ᵃ t-value is large. A Savage-Dickey approximation was used to compute the Bayes factor but no error estimate can be given. | | | | | |

**Main analyses**

Outcome neutral criteria were met in all criteria except for the BI in errors. Because we were mostly interested in RT, we ran the analyses.

Both hypotheses were directional but in opposite directions:

**For the TRCE**, we predicted that the TRCE will be reduced in the pro- compared to the anti- free will condition. This pattern was expected both in the control-prediction and in the automaticity prediction:

**Bayesian Independent Samples T-Test: TRCE**

| **Bayesian Independent Samples T-Test** | | | | | | | | | | | | | | | | | | | |
| --- | --- | --- | --- | --- | --- | --- | --- | --- | --- | --- | --- | --- | --- | --- | --- | --- | --- | --- | --- |
|  | | | | | | | | | | | | | **BF₀₊** | | | | | **error %** | |
| TRCE_mrt_effect | | | | | | | | | |  | | | 18.058 | | | |  | ~ 3.591e-4 |  |
| TRCE_effect_merr | | | | | | | | | |  | | | 25.323 | | | |  | ~ 8.958e-5 |  |
|  | | | | | | | | | | | | | | | | | | | |
| *Note.*  For all tests, the alternative hypothesis specifies that the location of group *anti* is greater than the location of group *pro* . | | | | | | | | | | | | | | | | | | | |
| **Descriptives** | | | | | | | | | | | | | | | | | | |  |
|  | | | | | | | | | | | | | | | **95% Credible Interval** | | | |  |
|  | | **Group** | | **N** | | **Mean** | | | **SD** | | | **SE** | | | **Lower** | | | **Upper** |  |
| TRCE_mrt_effect |  | anti |  | 268 |  | 363.945 |  | 370.412 | |  | 22.627 | |  | 319.396 | |  | 408.494 |  |  |
|  |  | pro |  | 299 |  | 371.635 |  | 369.986 | |  | 21.397 | |  | 329.527 | |  | 413.743 |  |  |
| TRCE_effect_merr |  | anti |  | 275 |  | 0.293 |  | 0.342 | |  | 0.021 | |  | 0.253 | |  | 0.334 |  |  |
|  |  | pro |  | 306 |  | 0.315 |  | 0.347 | |  | 0.020 | |  | 0.276 | |  | 0.354 |  |  |
|  | | | | | | | | | | | | | | | | | | |  |

**For the Bi & CRS effects**, we had two predictions. According to the control prediction, we predicted that the effects will be enhanced in the pro- compared to the anti- free will condition. According to the automaticity prediction, we predicted that it will not differ. We thus tested the directional hypothesis for both predictions:

**Bayesian Independent Samples T-Test: BI & CRS**

| **Bayesian Independent Samples T-Test** | | | | | | | | | | | | | | | | |
| --- | --- | --- | --- | --- | --- | --- | --- | --- | --- | --- | --- | --- | --- | --- | --- | --- |
|  | | | | | | | | | **BF₀₋** | | | | | | **error %** | |
| BI_mrt_effect | | | | | | | |  | 13.863 | | | | |  | ~ 5.682e-4 |  |
| BI_effect_merr | | | | | | | |  | 27.025 | | | | |  | ~ 1.447e-4 |  |
| crs_mrt_effect | | | | | | | |  | 7.457 | | | | |  | ~ 8.475e-4 |  |
| crs_effect_merr | | | | | | | |  | 14.918 | | | | |  | ~ 5.333e-4 |  |
|  | | | | | | | | | | | | | | | | |
| *Note.*  For all tests, the alternative hypothesis specifies that the location of group *anti* is smaller than the location of group *pro* . | | | | | | | | | | | | | | | | |
| **Descriptives** | | | | | | | | | | | | | | | |  |
|  | | | | | | | | | | | | **95% Credible Interval** | | | |  |
|  | | **Group** | | **N** | | **Mean** | | **SD** | | **SE** | | **Lower** | | **Upper** | |  |
| BI_mrt_effect |  | anti |  | 276 |  | 12.919 |  | 59.359 |  | 3.573 |  | 5.885 |  | 19.953 |  |  |
|  |  | pro |  | 310 |  | 13.517 |  | 63.941 |  | 3.632 |  | 6.371 |  | 20.662 |  |  |
| BI_effect_merr |  | anti |  | 276 |  | -3.099e-4 |  | 0.029 |  | 0.002 |  | -0.004 |  | 0.003 |  |  |
|  |  | pro |  | 310 |  | -0.002 |  | 0.031 |  | 0.002 |  | -0.006 |  | 9.782e-4 |  |  |
| crs_mrt_effect |  | anti |  | 266 |  | 36.695 |  | 99.248 |  | 6.085 |  | 24.714 |  | 48.677 |  |  |
|  |  | pro |  | 305 |  | 42.812 |  | 97.657 |  | 5.592 |  | 31.809 |  | 53.816 |  |  |
| crs_effect_merr |  | anti |  | 268 |  | 0.012 |  | 0.058 |  | 0.004 |  | 0.005 |  | 0.019 |  |  |
|  |  | pro |  | 306 |  | 0.012 |  | 0.050 |  | 0.003 |  | 0.007 |  | 0.018 |  |  |
|  | | | | | | | | | | | | | | | |  |

We did not reach boundaries, and therefore we continued with data collection.

**Correlations**

With FWI – only pro group:

Expecting a negative correlation with TRCE (test for a negative correlation):

**TRCE: Bayesian Correlation**

| **Bayesian Pearson Correlations** | | | | | | | | | |  |  |  |  |  |
| --- | --- | --- | --- | --- | --- | --- | --- | --- | --- | --- | --- | --- | --- | --- |
| **Variable** | |  | | **FWI** | |  | |  | |  | **------------------------------------------------------------------------------------------------------------------------------** |  |  |  |
| 1. FWI |  | n |  | — |  |  |  |  |  |  |  |  |  |  |
|  |  | Pearson's r |  | — |  |  |  |  |  |  |  |  |  |  |
|  |  | BF₀₋ |  | — |  |  |  |  |  |  |  |  |  |  |
| 2. TRCE_mrt_effect |  | n |  | 296.000 |  |  |  |  |  |  |  |  |  |  |
|  |  | Pearson's r |  | 0.050 |  |  |  |  |  |  |  |  |  |  |
|  |  | BF₀₋ |  | 24.190 |  |  |  |  |  |  |  |  |  |  |
| 3. TRCE_effect_merr |  | n |  | 303.000 |  |  |  |  |  |  |  |  |  |  |
|  |  | Pearson's r |  | 0.008 |  |  |  |  |  |  |  |  |  |  |
|  |  | BF₀₋ |  | 15.441 |  |  |  |  |  |  |  |  |  |  |
|  | | | | | | | | | |  |  |  |  |  |
| *Note.*  For all tests, the alternative hypothesis specifies that the correlation is negative | | | | | | | | | |  |  |  |  |  |

Expecting positive correlation with BI and CRS:

**BI & CRS: Bayesian Correlation**

| **Bayesian Pearson Correlations** | | | | | | | | | | | | | | | | |  |
| --- | --- | --- | --- | --- | --- | --- | --- | --- | --- | --- | --- | --- | --- | --- | --- | --- | --- |
| **Variable** | |  | | **FWI** | **-------------------** | | | **-------------------** | | | **-------------------** | | | **-------------------** | | |  |
| 1. FWI |  | n |  | — |  |  |  | |  |  | |  |  | |  |  | |
|  |  | Pearson's r |  | — |  |  |  | |  |  | |  |  | |  |  | |
|  |  | BF₀₊ |  | — |  |  |  | |  |  | |  |  | |  |  | |
| 2. BI_mrt_effect |  | n |  | 307.000 |  |  |  | |  |  | |  |  | |  |  | |
|  |  | Pearson's r |  | 0.019 |  |  |  | |  |  | |  |  | |  |  | |
|  |  | BF₀₊ |  | 10.508 |  |  |  | |  |  | |  |  | |  |  | |
| 3. BI_effect_merr |  | n |  | 307.000 |  |  |  | |  |  | |  |  | |  |  | |
|  |  | Pearson's r |  | 0.025 |  |  |  | |  |  | |  |  | |  |  | |
|  |  | BF₀₊ |  | 9.558 |  |  |  | |  |  | |  |  | |  |  | |
| 4. crs_mrt_effect |  | n |  | 303.000 |  |  |  | |  |  | |  |  | |  |  | |
|  |  | Pearson's r |  | 0.008 |  |  |  | |  |  | |  |  | |  |  | |
|  |  | BF₀₊ |  | 12.420 |  |  |  | |  |  | |  |  | |  |  | |
| 5. crs_effect_merr |  | n |  | 304.000 |  |  |  | |  |  | |  |  | |  |  | |
|  |  | Pearson's r |  | 0.115 |  |  |  | |  |  | |  |  | |  |  | |
|  |  | BF₀₊ |  | 0.983 |  |  |  | |  |  | |  |  | |  |  | |
|  | | | | | | | | | | | | | | | | |  |
| *Note.*  For all tests, the alternative hypothesis specifies that the correlation is positive. | | | | | | | | | | | | | | | | |  |

Entire sample, FWI:

Expecting a negative correlation with TRCE (test for a negative correlation):

**FWI - Bayesian Correlation: TRCE**

| **Bayesian Pearson Correlations** | | | | | | | | | |
| --- | --- | --- | --- | --- | --- | --- | --- | --- | --- |
| **Variable** | |  | | **FWI** | | **_____ _______** | | **____________** | |
| 1. FWI |  | n |  | — |  |  |  |  |  |
|  |  | Pearson's r |  | — |  |  |  |  |  |
|  |  | BF₀₋ |  | — |  |  |  |  |  |
| 2. TRCE_mrt_effect |  | n |  | 562.000 |  |  |  |  |  |
|  |  | Pearson's r |  | 0.029 |  |  |  |  |  |
|  |  | BF₀₋ |  | 30.437 |  |  |  |  |  |
| 3. TRCE_effect_merr |  | n |  | 574.000 |  |  |  |  |  |
|  |  | Pearson's r |  | 0.056 |  |  |  |  |  |
|  |  | BF₀₋ |  | 43.226 |  |  |  |  |  |
|  | | | | | | | | | |
| *Note.*  For all tests, the alternative hypothesis specifies that the correlation is negative | | | | | | | | | |

Expecting positive correlation with BI and CRS:

**FWI - Bayesian Correlation: BI & CRS**

| **Bayesian Pearson Correlations** | | | | | | | | | | | | | |
| --- | --- | --- | --- | --- | --- | --- | --- | --- | --- | --- | --- | --- | --- |
| **Variable** | |  | | **FWI** | | **------------** | | **------------** | | **------------** | | **------------** | |
| 1. FWI |  | n |  | — |  |  |  |  |  |  |  |  |  |
|  |  | Pearson's r |  | — |  |  |  |  |  |  |  |  |  |
|  |  | BF₀₊ |  | — |  |  |  |  |  |  |  |  |  |
| 2. BI_mrt_effect |  | n |  | 579.000 |  |  |  |  |  |  |  |  |  |
|  |  | Pearson's r |  | 0.013 |  |  |  |  |  |  |  |  |  |
|  |  | BF₀₊ |  | 14.621 |  |  |  |  |  |  |  |  |  |
| 3. BI_effect_merr |  | n |  | 579.000 |  |  |  |  |  |  |  |  |  |
|  |  | Pearson's r |  | -0.005 |  |  |  |  |  |  |  |  |  |
|  |  | BF₀₊ |  | 20.905 |  |  |  |  |  |  |  |  |  |
| 4. crs_mrt_effect |  | n |  | 568.000 |  |  |  |  |  |  |  |  |  |
|  |  | Pearson's r |  | 0.025 |  |  |  |  |  |  |  |  |  |
|  |  | BF₀₊ |  | 11.011 |  |  |  |  |  |  |  |  |  |
| 5. crs_effect_merr |  | n |  | 571.000 |  |  |  |  |  |  |  |  |  |
|  |  | Pearson's r |  | 0.103 |  |  |  |  |  |  |  |  |  |
|  |  | BF₀₊ |  | 0.474 |  |  |  |  |  |  |  |  |  |
|  | | | | | | | | | | | | | |
| *Note.*  For all tests, the alternative hypothesis specifies that the correlation is positive. | | | | | | | | | | | | | |

Entire sample, LOC, no clear predictions:

**LoC: Bayesian Correlation - Exploratory**

| **Variable** | |  | | **LoC** | |  |
| --- | --- | --- | --- | --- | --- | --- |
| 1. LoC |  | n |  | — |  |  |
|  |  | Pearson's r |  | — |  |  |
|  |  | BF₀₁ |  | — |  |  |
| 2. FWI |  | n |  | 581.000 |  |  |
|  |  | Pearson's r |  | 0.419 |  |  |
|  |  | BF₀₁ |  | 1.161e-23 |  |  |
| 3. TRCE_mrt_effect |  | n |  | 565.000 |  |  |
|  |  | Pearson's r |  | -0.001 |  |  |
|  |  | BF₀₁ |  | 18.967 |  |  |
| 4. TRCE_effect_merr |  | n |  | 577.000 |  |  |
|  |  | Pearson's r |  | 0.020 |  |  |
|  |  | BF₀₁ |  | 17.041 |  |  |
| 5. BI_mrt_effect |  | n |  | 582.000 |  |  |
|  |  | Pearson's r |  | 0.081 |  |  |
|  |  | BF₀₁ |  | 2.939 |  |  |
| 6. BI_effect_merr |  | n |  | 582.000 |  |  |
|  |  | Pearson's r |  | -0.027 |  |  |
|  |  | BF₀₁ |  | 15.520 |  |  |
| 7. crs_mrt_effect |  | n |  | 571.000 |  |  |
|  |  | Pearson's r |  | 0.025 |  |  |
|  |  | BF₀₁ |  | 15.978 |  |  |
| 8. crs_effect_merr |  | n |  | 574.000 |  |  |
|  |  | Pearson's r |  | 0.034 |  |  |
|  |  | BF₀₁ |  | 13.657 |  |  |

**Pre-registered regressions**

**TRCE-RT Bayesian Linear Regression - LoC**

| **Model Comparison - TRCE_mrt_effect** | | | | | | | | | | | |
| --- | --- | --- | --- | --- | --- | --- | --- | --- | --- | --- | --- |
| **Models** | | **P(M)** | | **P(M\|data)** | | **BF_M_** | | **BF_10_** | | **R²** | |
| Null model |  | 0.333 |  | 0.902 |  | 18.454 |  | 1.000 |  | 0.000 |  |
| cond_pro0.5antiminus0.5 |  | 0.167 |  | 0.043 |  | 0.225 |  | 0.095 |  | 0.000 |  |
| LoC |  | 0.167 |  | 0.042 |  | 0.220 |  | 0.094 |  | 0.000 |  |
| cond_pro0.5antiminus0.5 + LoC |  | 0.333 |  | 0.013 |  | 0.025 |  | 0.014 |  | 0.000 |  |
|  | | | | | | | | | | | |

**TRCE-PE Bayesian Linear Regression - LoC**

| **Model Comparison - TRCE_effect_merr** | | | | | | | | | | | |
| --- | --- | --- | --- | --- | --- | --- | --- | --- | --- | --- | --- |
| **Models** | | **P(M)** | | **P(M\|data)** | | **BF_M_** | | **BF_10_** | | **R²** | |
| Null model |  | 0.333 |  | 0.865 |  | 12.824 |  | 1.000 |  | 0.000 |  |
| cond_pro0.5antiminus0.5 |  | 0.167 |  | 0.069 |  | 0.372 |  | 0.160 |  | 0.002 |  |
| LoC |  | 0.167 |  | 0.045 |  | 0.235 |  | 0.104 |  | 0.000 |  |
| cond_pro0.5antiminus0.5 + LoC |  | 0.333 |  | 0.021 |  | 0.042 |  | 0.024 |  | 0.002 |  |
|  | | | | | | | | | | | |

**BI-RT Bayesian Linear Regression - LoC**

| **Model Comparison - BI_mrt_effect** | | | | | | | | | | | |
| --- | --- | --- | --- | --- | --- | --- | --- | --- | --- | --- | --- |
| **Models** | | **P(M)** | | **P(M\|data)** | | **BF_M_** | | **BF_10_** | | **R²** | |
| Null model |  | 0.333 |  | 0.702 |  | 4.720 |  | 1.000 |  | 0.000 |  |
| LoC |  | 0.167 |  | 0.204 |  | 1.283 |  | 0.582 |  | 0.006 |  |
| cond_pro0.5antiminus0.5 + LoC |  | 0.333 |  | 0.061 |  | 0.130 |  | 0.087 |  | 0.007 |  |
| cond_pro0.5antiminus0.5 |  | 0.167 |  | 0.032 |  | 0.167 |  | 0.092 |  | 0.000 |  |
|  | | | | | | | | | | | |

**CRS-RT Bayesian Linear Regression - LoC**

| **Model Comparison - crs_mrt_effect** | | | | | | | | | | | |
| --- | --- | --- | --- | --- | --- | --- | --- | --- | --- | --- | --- |
| **Models** | | **P(M)** | | **P(M\|data)** | | **BF_M_** | | **BF_10_** | | **R²** | |
| Null model |  | 0.333 |  | 0.881 |  | 14.746 |  | 1.000 |  | 0.000 |  |
| cond_pro0.5antiminus0.5 |  | 0.167 |  | 0.054 |  | 0.283 |  | 0.122 |  | 0.001 |  |
| LoC |  | 0.167 |  | 0.049 |  | 0.256 |  | 0.111 |  | 0.001 |  |
| cond_pro0.5antiminus0.5 + LoC |  | 0.333 |  | 0.017 |  | 0.035 |  | 0.019 |  | 0.001 |  |
|  | | | | | | | | | | | |

**CRS-PE Bayesian Linear Regression - LoC**

| **Model Comparison - crs_effect_merr** | | | | | | | | | | | |
| --- | --- | --- | --- | --- | --- | --- | --- | --- | --- | --- | --- |
| **Models** | | **P(M)** | | **P(M\|data)** | | **BF_M_** | | **BF_10_** | | **R²** | |
| Null model |  | 0.333 |  | 0.885 |  | 15.431 |  | 1.000 |  | 0.000 |  |
| LoC |  | 0.167 |  | 0.057 |  | 0.303 |  | 0.129 |  | 0.001 |  |
| cond_pro0.5antiminus0.5 |  | 0.167 |  | 0.041 |  | 0.214 |  | 0.093 |  | 0.000 |  |
| cond_pro0.5antiminus0.5 + LoC |  | 0.333 |  | 0.017 |  | 0.034 |  | 0.019 |  | 0.001 |  |
|  | | | | | | | | | | | |

**Further t-tests**

The impact the manipulation had on: FWI (free will inventory, LoC (locus of control), subjective fatigue, and RT (mrt).

**Impact of the manipulation on the FWI manipulation**

## Bayesian Independent Samples T-Test: FWI

| **Bayesian Independent Samples T-Test** | | | | | | | | | | | | |  |  |  |
| --- | --- | --- | --- | --- | --- | --- | --- | --- | --- | --- | --- | --- | --- | --- | --- |
|  | | | **BF₁₀** | | | | | | **error %** | | | |  |  |  |
| FWI | |  | 9.417e+14 | | | | |  | 3.980e-20 | | |  |  |  |  |
|  | | | | | | | | | | | | |  |  |  |
| **Descriptives** | | | | | | | | | | | | | | | |
|  | | | | | | | | | | | | **95% Credible Interval** | | | |
|  | | **Group** | | **N** | | **Mean** | | **SD** | | **SE** | | **Lower** | | **Upper** | |
| FWI |  | anti |  | 272 |  | 3.825 |  | 1.196 |  | 0.073 |  | 3.682 |  | 3.968 |  |
|  |  | pro |  | 309 |  | 4.723 |  | 1.214 |  | 0.069 |  | 4.587 |  | 4.859 |  |
|  | | | | | | | | | | | | | | | |

## Impact on fatigue & LoC: Bayesian Independent Samples T-Test: Fatigue & LoC

| **Bayesian Independent Samples T-Test** | | | | | | | | |  |  |  |  |  |  |  |
| --- | --- | --- | --- | --- | --- | --- | --- | --- | --- | --- | --- | --- | --- | --- | --- |
|  | | | **BF₀₁** | | **error %** | | | |  |  |  |  |  |  |  |
| subjective_fatigue | |  | 13.297 |  | 0.001 | | |  |  |  |  |  |  |  |  |
| mrt | |  | 14.983 |  | 0.001 | | |  |  |  |  |  |  |  |  |
| LoC | |  | 0.002 |  | 1.607e-7 | | |  |  |  |  |  |  |  |  |
|  | | | | | | | | |  |  |  |  |  |  |  |
| **Descriptives** | | | | | | | | | | | | | | | |
|  | | | | | | | | | | | | **95% Credible Interval** | | | |
|  | | **Group** | | **N** | | **Mean** | | **SD** | | **SE** | | **Lower** | | **Upper** | |
| subjective_fatigue |  | anti |  | 273 |  | 3.566 |  | 1.172 |  | 0.071 |  | 3.426 |  | 3.706 |  |
|  |  | pro |  | 311 |  | 3.515 |  | 1.168 |  | 0.066 |  | 3.385 |  | 3.646 |  |
| mrt |  | anti |  | 276 |  | 976.032 |  | 316.869 |  | 19.073 |  | 938.483 |  | 1013.580 |  |
|  |  | pro |  | 312 |  | 981.265 |  | 329.309 |  | 18.643 |  | 944.582 |  | 1017.949 |  |
| LoC |  | anti |  | 273 |  | 8.121 |  | 3.938 |  | 0.238 |  | 7.652 |  | 8.590 |  |
|  |  | pro |  | 311 |  | 9.598 |  | 4.406 |  | 0.250 |  | 9.106 |  | 10.090 |  |
|  | | | | | | | | | | | | | | | |

1. Due to a coding error, we ran 93 participants in the anti-free will condition after Run 1. After the error was discovered, we decided not to analyze it, and continued as originally planned. The data was not analyzed but it is available in OSF. [↑](#footnote-ref-1)
